# Geographical origin identification of Chinese white teas, and their differences in tastes, chemical compositions and antioxidant activities among three production regions

**DOI:** 10.1016/j.fochx.2022.100504

**Published:** 2022-11-07

**Authors:** Cunqiang Ma, Bingsong Ma, Jiacai Wang, Zihao Wang, Xuan Chen, Binxing Zhou, Xinghui Li

**Affiliations:** a College of Horticulture, Nanjing Agricultural University, Nanjing, 210095, Jiangsu, China; b College of Tea, Yunnan Agricultural University, Kunming 650201, Yunnan, China; c Henan Key Laboratory of Tea Comprehensive Utilization in South Henan, Tea College, Xinyang Agriculture and Forestry University, Xinyang 464000, Henan, China; d Menghai Branch, Zhongcha Tea Industry Co. Ltd, Xishuangbanna 666200, Yunnan, China

**Keywords:** C, (+)-catechin, EC, (−)-epicatechin, ECG, (−)-epicatechin gallate, GCG, (−)-gallocatechin gallate, EGCG, (−)-epigallocatechin gallate, EGC, (−)-epigallocatechin, VOCs, volatile organic compounds, PCA, principal component analysis, OPLS-DA, orthogonal partial least squares-discriminant analysis, HPLC, high-performance liquid chromatography, LC-MS, liquid chromatography-mass spectrometry, GABA, γ-aminobutyric acid, E-tongue, electronic tongue, GC, (+)-gallocatechin, CG, (−)-catechin gallate, TGG, 1,4,6-tri-*O*-galloyl-β-d-glucose, CNIS, China National Institute of Standardization, IEC, ion-exchange chromatography, HCA, hierarchical cluster analysis, PLS-DA, partial least squares-discriminant analysis, UV–vis, ultraviolet–visible, ANOVA, analysis of variance, VIP, variable importance in the projection, Gallic acid (PubChem CID 370), Theaflavin (PubChem CID 135403798), L-glutamic acid (PubChem CID 33032), Theaflavin-3-gallate (PubChem CID 136825044), Theaflavine-3,3′-digallate (PubChem CID 3589471), (−)-Epicatechin gallate (PubChem CID 107905), Quercetin (PubChem CID 5280459), Kaempferol (PubChem CID 5280863), L-theanine (PubChem CID 439378), Luteolin (PubChem CID 5280445), White tea, Production region, Multivariate statistical analysis, Catechins, Umami

## Abstract

The producing area of Chinese white tea has been expanded to Xinyang and Yunnan from Fuding region. In this study, six sensory tastes and fifty-one chemical components including seventeen phenolic compounds, three purine alkaloids and twenty amino acids were determined in eighteen Bai mudan sub-type of white tea by electronic tongue, high performance liquid chromatography and amino acid analyzer for geographical identification, respectively. Additionally, *in vitro* antioxidant activities were evaluated by five various assays. Multivariate statistical analyses such as PCA, HCA and PLS-DA, completely divided these white teas into Xinyang, Yunnan and Fuding groups, indicating the feasibility of white tea classification by the production region. Twelve characteristic compounds (VIP > 1.0, *P* < 0.05) like gallic acid, theaflavin and L-glutamic acid contributed to the geographical identification. In conclusion, this study explored the chemical, taste and antioxidant capacity differences among three main production regions, and revealed their potential relations in white tea.

## Highlights

• These white teas completely divided into three groups by production region via chemometrics.
• 12 Characteristic compounds (VIP > 1.0, *P* < 0.05) contributed to geographical identification.
• Yunnan white tea had highest 6 catechins, 4 flavonoids, 2 purine alkaloids, antioxidant ability.
• Theaflavins, caffeine, gallic acid, catechins, amino acids significantly impact the taste.
• 5 Catechins (*P* < 0.001, r > 0.50) such as EGCG regarded as the antioxidants in Chinese white tea.

## Introduction

As lightly fermented tea because of the partial preservation of endogenous polyphenol oxidase and peroxidase in the processing, white tea is mainly produced from fresh tea-leaves (*Camellia* si*nensis* L.) through prolonged withering and drying (Yang, Baldermann, & Watanabe, 2013). Chen et al., 2019, Chen et al., 2020 have explored the dynamic changes of volatile and non-volatile compounds during the whole processing, and found its remarkable influence on phenolic compounds including catechins, flavonoids, phenolic acids and their glucoside, caffeine and amino acids, for instances, the significant (*P* < 0.05) decreases of catechins such as (+)-catechin (C), (−)-epicatechin (EC), (−)-epicatechin gallate (ECG), (−)-gallocatechin gallate (GCG) and (−)-epigallocatechin gallate (EGCG) in the prolonged withering (Wang et al., 2019, Zhao et al., 2019). Compared with the green tea and other teas, our previous study (Zhou, Wang et al., 2022) have confirmed the significantly (*P* < 0.05) higher contents of gallic acid, (−)-epigallocatechin (EGC) and several flavonoids (i.e. quercetin, myricetin, taxifolin and luteolin) in white tea, because of the hydrolyzation and oxidation of ester catechins and flavonoid glucosides during the prolonged withering.

Owing to the multiple health benefits of Chinese white tea (Sanlier, Atik, & Atik, 2018), such as antioxidant (Dias et al., 2014), anti-inflammatory, anti-mutagenic, antitumor (Liu et al., 2018) and neuroprotective activities (Pastoriza, Mesías, Cabrera, & Rufián-Henares, 2017), its producing area has been expanded from Fuding Region, the Northeast of Fujian Province to Xinyang Region of Henan Province, Lincang and Puer Region of Yunnan Province in China with three primary sub-types, such as Baihaoyinzhen (Silver Needle), Bai mudan (White Peony) and Shou Mei (Long Brow) (Yang et al., 2018). Additionally, D. Zhang et al. (2020) has accomplished the rapid and direct origin identification of white tea in Fujian Province based on volatile organic compounds (VOCs). However, the characteristic components for the geographical identification at a national scale have not been revealed yet, including the exploration about their taste, chemical and antioxidant activities differences among the major production areas of Chinese white tea.

At present, high-performance liquid chromatography (HPLC) (Wang et al., 2014), amino acid analyzer (Ma et al., 2021) and liquid chromatography-mass spectrometry (LC-MS)-based widely targeted metabonomics (Wang, Gan, Sun, & Chen, 2022) have been developed for the determinations of phenolic compounds, purine alkaloids, amino acids and other quality components, which could achieve the geographical identification of green tea (Wang, Ma et al., 2021), black tea (Fang et al., 2019) and oolong tea through principal component analysis (PCA), orthogonal partial least squares-discriminant analysis (OPLS-DA) and partial least squares-discriminant analysis (PLS-DA) (Meng et al 2017). In this work, eighteen Bai mudan sub-type of white teas were collected from various production regions for the quantitative determinations of phenolic compounds, purine alkaloids and amino acids by HPLC and amino acid analyzer to select the characteristic compounds for geographical identification of Chinese white teas, respectively. Additionally, electronic tongue (E-tongue) and five various assays were carried out for the objective evaluation of sensory tastes and the *in vitro* antioxidant activities. Combined with the multivariate statistical analyses such as PCA, hierarchical cluster analysis (HCA) and PLS-DA, as well as univariate statistical analysis, this study might achieve the geographical origin identification of Chinese white teas according to their tastes or chemical differences, and reveal their potential correlations among tastes, chemical compositions and antioxidant activities in white tea.

## Materials and methods

### Materials and reagents

The eighteen Bai Mudan sub-type of commercial white tea samples collected from Xinyang Region of Henan Province (named as XYT-1 to XYT-6), Fuding Region of Fujian Province (named as FDT-1 to FDT-6), and Lincang and Puer Regions of Yunnan Province (named as YNT-1 to YNT-6), were all made by the fresh tea-leaves with one bud and two leaves of the locally grown varieties/cultivars, such as Xinyang group (*Camellia* si*nensis* var. *sinensis* cv. Xinyang group), Fuding Dabaicha (*Camellia* si*nensis* var. *sinensis* cv. Fuding Dabaicha), Fuding Dahaocha (*Camellia* si*nensis* var. *sinensis* cv. Fuding Dahaocha), Jinggu Dabaicha (*Camellia sinensis* var. *assamica* cv. Jinggu Dabaicha) and Mengku Dayezhong (*Camellia sinensis* var. *assamica* cv. Mengku Dayezhong) in the spring season of 2020, according to local processing technology, which have been listed in Table S1. All processed tea samples were maintained at −20 °C with a moisture content below 6.5% for sensory evaluation, and tea powders were collected through 40 mesh filtration for chemical determination and antioxidant capacity test.

Eight catechins, i.e., C, EC, EGC, ECG, GCG, EGCG, (+)-gallocatechin (GC) and (−)-catechin gallate (CG), two phenolic acids including gallic acid and ellagic acid, as well as 1,4,6-tri-*O*-galloyl-β-d-glucose (TGG) (purity ≥ 98.0%) were purchased from Yuanye Bio-Technology Co., ltd (Shanghai, China). Six flavonoids standards, i.e., quercetin, kaempferol, myricetin, taxifolin, luteolin and rutin were purchased from Must Bio-Technology Co., Ltd (Chengdu, Sichuan, China) with a purity ≥ 98.0%. Three purine alkaloids (i.e. caffeine, theobromine and theophylline), four theaflavins (i.e. theaflavin, theaflavin-3-gallate, theaflavin 3,3′-digallate and theaflavin-3′-gallate), nineteen amino acids (i.e l-asparagine, l-alanine, l-aspartic acid, l-argnine, l-cysteine, l-glutamic acid, l-glycine, l-histidine, l-isoleucine, l-leucine, l-methionine, l-phenylalanine, l-proline, l-theanine, l-threonine, l-tryptophane, l-tyrosine, l-serine and l-valine) and GABA were purchased from Sigma-Aldrich Co., ltd (St. Louis, MO, USA) with a purity no<98.0%. Chromatographic-grade acetonitrile, methanol, 2-methoxyethanol and acetic acid were purchased from Aladdin Biological Co., Ltd (Shanghai, China).

### Sensory evaluation

According to China National Institute of Standardization (CNIS) GB/T 23776-2018, seven panelists were randomly selected from ten professional tea tasters for objective sensory evaluations of the appearance (a), liquor color (b), aroma (c), and taste (d) and tea-leaves residues (e). The detailed approaches are described in Text S1 in the supplemental material.

Total score was estimated as follows:

Total score = 20% × appearance (a) + 10% × liquor color (b) + 30% × aroma (c) + 30% × taste (d) + 10% × tea-leaves residues (e).

### E-tongue for taste evaluation

An E-tongue (SA402B, INSENT, Japan) composed of the six taste sensors (i.e.1-AAE, 2-CTO, 3-CAO, 5-COO, 6-AE1 and 5-GL1) and reference electrode (R), was developed for objective evaluation of umami, saltiness, sourness, bitterness, astringency and sweetness in white teas infusion with uniform proportion of 1:50 (g/mL). After extraction of boiling water for 5 min, the tea infusion was cooled to indoor temperature (25 °C) for E-tongue detection, which have been described in the reports of Xu et al., 2019, Ma et al., 2022 and Text S1 in the supplemental material, respectively.

### Determinations of seven main quality components in white teas

The moisture content and water extracts content were measured in accordance with the CNIS GB 5009.3-2016 and GB/T 8305-2013 methods, respectively. Seven major quality components, i.e., tea polyphenols, total flavonoids, soluble-sugars, free amino acids, theaflavins, thearubigins and theabrownins, were determined by a TU-1901 ultraviolet–visible (UV–vis) spectrophotometer (Puxi Technologies, Beijing, China) according to the CNIS or the established methods in previous studies (Wang et al., 2021, Tong et al., 2019, Zhou et al., 2022). For example, the total content of free amino acids was measured on 570 nm by the UV–vis spectrophotometer through ninhydrin assay with the l-glutamic acid for the standard curves (Zhou, Ma et al., 2022). The detailed approaches of UV–vis spectrophotometer also have been described in Text S1 in the supplemental material.

### Seventeen phenolic compounds and three purine alkaloids contents determined by HPLC

The seventeen phenolic compounds including eight catechins, six flavonoids, two phenolic acids and TGG, and three purine alkaloids contents in eighteen white tea samples were determined by the established and evaluated HPLC method (Nian et al., 2019), using an Agilent 1200 series HPLC system (Agilent Technologies, Santa Clara, CA, USA) comprised of a Poroshell 120 EC-C_18_ chromatogram column (100 × 4.6 mm, 2.7 μm; Agilent Technologies, Santa Clara, CA, USA) and a C_18_ guard column (10 × 4.6 mm, 5 μm; Phenomenex, Torrance, CA, USA) with solvent A (5% acetonitrile and 0.261% *ortho*-phosphoric acid water solution) and solvent B (80% methanol solution) as mobile phases. The detailed approaches of tea extraction and HPLC separation are described in Text S1 in the supplemental material. The linear regression equations of standard curve (Table S2) with a high correlation coefficient (R^2^ > 0.990) were established for the quantitative determination.

### Four theaflavins in white tea determined by HPLC

The 200 mg tea powder was extracted with 5 mL 70% (v/v) methanol solution at 70 °C for 10 min. Repeat extraction was carried out to obtain a metered volume of 10 mL after centrifugation at 3500 rpm for 5 min. After filtration by 0.45 μm nylon membrane, 5 μL filter liquor was injected into Agilent 1100VL series HPLC system (Agilent Technologies, Santa Clara, CA, USA) comprised of an Agilent SB-Aq C18 reversed-phase chromatogram column (250 × 4.6 mm, 5 μm) for the quantitative analysis of the four theaflavins. The solvent A (90 mL acetonitrile and 20 mL acetic acid with 20 mg EDTA-2Na in 1000 mL water solution) and solvent B (800 mL methanol and 20 mL acetic acid with 20 mg EDTA-2Na in 1000 mL water solution) were prepared as the mobile phases for the HPLC separation. The detailed approaches are described in Text S1 in the supplemental material. The linear regression equations of standard curve (R^2^ > 0.999) were established to calculate the quantitative contents of four theaflavins (Table S3).

### Nineteen amino acids and GABA contents determined by amino acid analyzer

A S-433D amino acid analyzer (Sykam Technologies, Munich, Bavaria, Germany) with a LCA K07/Li cation separation column (150 mm × 4.6 mm, 3 μm) using ion-exchange chromatography (IEC) was carried out to determine the nineteen amino acids and GABA contents in each white tea sample after ninhydrin derivations, which has been described in our previous studies of Ma et al., 2021, Zhou et al., 2022. The detailed approaches are described in Text S1 in the supplemental material. The contents of nineteen amino acids and GABA in eighteen white tea samples were calculated through the linear regression equations (Table S4) with a high correlation coefficient (R^2^ > 0.990).

### *In vitro* antioxidant capacity evaluated by five various assays

The five various assays including ferric ion reducing antioxidant power (FRAP), DPPH free radical scavenging activity (DPPH), ABTS^•+^ scavenging activity (ABTS), hydroxyl radical scavenging ability (HSA) and superoxide anion radical scavenging ability (SSA), were developed for the evaluation of *in vitro* antioxidant capacity in each white tea, respectively, as described in the report of Ma et al. (2022). Specifically, the 50 mg tea powder mixed with 1 mL 80% (v/v) ethanol water was extracted by the ultrasonic producer for the FRAP detection. The 50 mg tea powder mixed with 1 mL 80% (v/v) methanol solution was extracted by tissue homogenate in an ice bath for DPPH and ABTS detection. The 100 mg tea powder was extracted by tissue homogenate in an ice bath with 1 mL distilled water for the HSA detection. The 100 mg tea power was extracted by tissue homogenate in an ice bath with 1 mL extracting solution for the SSA detection. The detailed approaches are described in Text S1 in the supplemental material.

### Statistical analysis

Three replications were executed to obtain the data present by mean value ± standard deviation. PCA, hierarchical cluster analysis (HCA) and PLS-DA were performed with fifty-four selected objects (18 × 3) by Origin 9.0 software (Hampton, MA, USA). The One-way analysis of variance (ANOVA) using Duncan s multiple range test and the independent-samples *t*-test to explore tastes, chemical and antioxidant activity differences, as well as the bivariate correlation analysis for the Person correlation coefficient to analyze their potential connections, were performed with IBM SPSS 20.0 software (Chicago, IL, USA). The relevant heat maps were performed by Origin 9.0 software (Hampton, MA, USA). The characteristic components were selected with a variable importance in the projection over 1.0 (VIP > 1.0) in PLS-DA and *P*-value below 0.05 (P < 0.05) in ANOVA for the geographical identification of Chinese white teas.

## Results and discussion

### Origin identification of white teas based on six sensory tastes

**Fig. 1:**
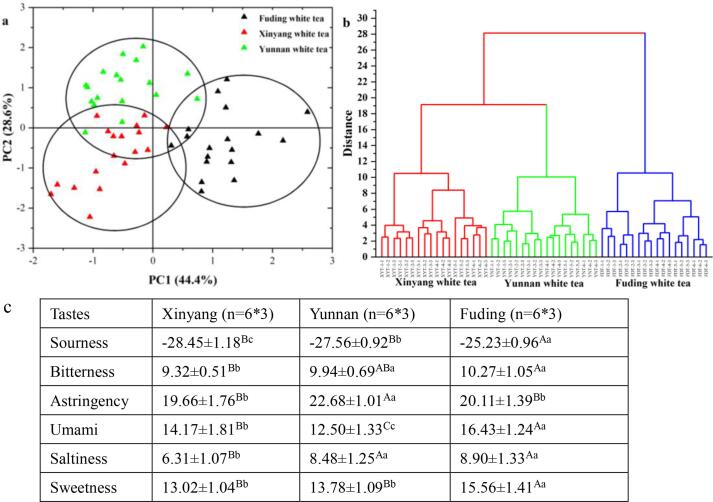
E-tongue results of white teas and their difference among three production regions.

E-tongue has been developed for the qualitative and quantitative assessment of tea taste (Xu et al., 2019), which could achieve the geographical identification of Chinese white tea via PCA (Fig. 1a) and HCA (Fig. 1b), and elaborate their tastes differences through one-way ANOVA (Fig. 1c). In the PCA (Fig. 1a), the first two principal components (PC1 = 43.7%, PC2 = 25.4%), elaborating 69.1% of total variance, basically divide these white teas into Xinyang, Yunnan and Fuding groups, which was consistent with HCA (Fig. 1b) result that the white teas from same origin were clustered together as a group in accordance with the production region. Therefore, the E-tongue results revealed that Chinese white teas could be classified by the production region at a national scale, such as Xinyang white tea, Yunnan white tea and Fuding white tea.

The one-way ANOVA (Fig. 1c) indicated the highly significant (*P* < 0.01) differences of six tastes among three production regions, and confirmed the highest sourness, bitterness, umami, saltiness and sweetness in Fuding white tea. Conversely, the Yunnan white tea showed a highly significantly (*P* < 0.01) higher astringency value than Fuding or Xinyang white teas, which might be attributed to the difference in distribution and contents of phenolic compounds. Generally, the E-tongue results were consistent with the traditional sensory evaluation in taste (Tables S5–S7). Furthermore, the traditional sensory evaluation also indicated the significant (*P* < 0.05) differences of aroma, shape and liquor color in white tea among three production regions, and confirmed the excellent quality of Fuding white tea, because of its relatively long history and advanced manufacturing technology. However, traditional sensory evaluation depended on professional tea tasters and their subjective judgment, which limited its application in the geographical identification.

### Origin identification of Chinese white teas through multivariate statistical analyses

**Fig. 2:**
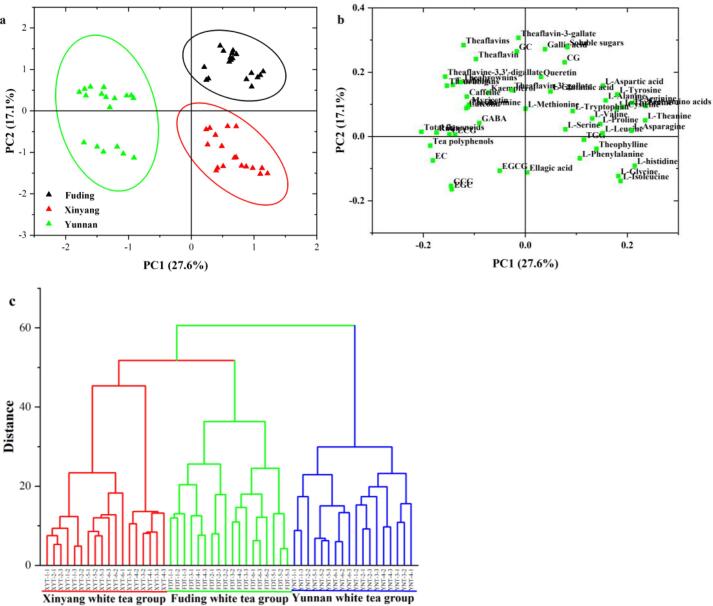
Principal component analysis (PCA, A and B) and hierarchical cluster analysis (HCA, C) divided these spring white teas into three groups (i.e. Xinyang, Yunnan and Fuding) followed by the production regions.

**Fig. 3:**
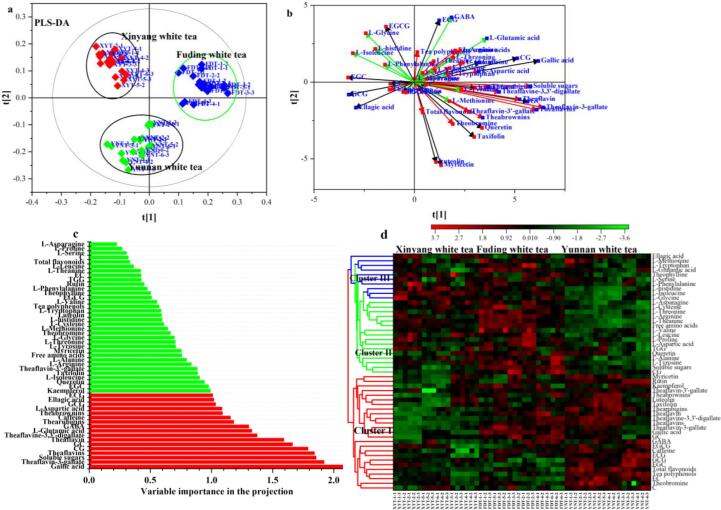
Contributions of chemical components to the geographical identification of white teas and their distributions among three production regions (i.e. Xinyang, Fuding and Yunnan).

In this study, based on fifty-one chemical components including seven main quality components, seventeen phenolic compounds, three purine alkaloids, four theaflavins, nineteen amino acids and GABA determined by UV–vis spectrophotometer, HPLC and amino acid analyzer, the multivariate statistical analyses (Fig. 2 and Fig. 3), such as PCA (Fig. 2a and b), HCA (Fig. 2c) and PLS-DA (Fig. 3a and b), could completely divide these white teas into Xinyang, Yunnan and Fuding white tea groups, respectively, which indicated the feasibility of Chinese white teas classification by production regions. The first two principal components (PC1 = 27.6% and PC2 = 17.1%) in the PCA (Fig. 2a) explained about 47.7% of total variability, and divided these white tea samples into three groups, i.e., Xinyang white tea, Fuding white tea and Yunan white tea groups. Generally, Yunnan white teas were observed to possess relatively higher catechins, purine alkaloids, tea pigments and flavonoids levels, such as EC, EGC, GCG, ECG, rutin, taxifolin, myricetin, thearubigins, theabrownins, theaflavine-3,3′-digallate, caffeine, theobromine and GABA, while Fuding white teas contained relatively higher gallic acid, soluble sugars and amino acids, such as l-theanine, l-arginine, l-aspartic acid, l-asparagine, l-arginine and l-glutamic acid (Fig. 2b). Additionally, Xinyang white tea comprised relatively higher l-histidine, l-isoleucine, l-glycine and l-phenylalanine. The HCA (Fig. 2c) also achieved the classification of white teas by their production regions, i.e. Xinyang white tea, Yunnan white tea and Fuding white tea. The same variety belonging to (*Camellia* si*nensis* var. si*nensis*) promoted that Fuding and Xinyang could be clustered as one subgroup, which showed certain chemical differences from Yunnan white tea produced from the fresh tea-leaves of (*Camellia sinensis* var. *assamica*).

Combined with HPLC, amino acid analyzer or LC-MS determination, PLS-DA has realized the classification of black tea according to origin, processing (i.e. degree of enzymatic fermentation) and botanical varieties (Shevchuk et al., 2018, Zhou et al., 2022). In this study, the PLS-DA model (Fig. 3a and b) obtain an excellent result of the discrimination in the origin of white tea (R^2^Y = 0.891 and Q^2^ = 0.862, respectively) at a relatively higher prediction accuracy (*P* < 0.001). Additionally, twelve characteristic components with a variable importance in the projection over 1.0 (VIP > 1.0) in PLS-DA model (Fig. 3d) and *P*-value below 0.05 (*P* < 0.05) through ANOVA (Table 1), including four catechins (i.e. ECG, GCG, GC and CG), gallic acid, three theaflavins (i.e. theaflavin, theaflavin-3-gallate and theaflavine-3,3′-digallate), l-glutamic acid, l-aspartic acid, GABA and caffeine, contributed to the geographical identification of Chinese white teas.

**Table 1:** Differences of seven main quality components, seventeen phenolic compounds, three purine alkaloids, four theaflavins, nineteen amino acids and GABA contents in white tea among Xinyang, Fuding and Yunnan regions.

Components (mg/g)	Production regions			ANOVA	
	Xinyang (n = 6 × 3)	Yunan (n = 6 × 3)	Fuding (n = 6 × 3)	*F*-value	*P*-value
Tea polyphenols	206.71 ± 18.38^Bb^	260.81 ± 12.97^Aa^	217.47 ± 18.88^Bb^	51.357	<0.05
Total flavonoids	24.39 ± 1.43^Bb^	33.80 ± 3.37^Aa^	24.54 ± 4.11^Bb^	51.812	<0.05
Free amino acids	28.39 ± 2.29^Bb^	22.09 ± 1.85^Cc^	32.37 ± 2.63^Aa^	93.132	<0.05
Soluble sugars	45.74 ± 5.38^Bc^	48.68 ± 4.14^Bb^	60.67 ± 2.91^Aa^	61.961	<0.05
Total catechins	125.79 ± 15.82^Bb^	150.32 ± 12.83^Aa^	122.87 ± 4.67^Bb^	28.110	<0.05
Theaflavins	2.87 ± 0.39^Ab^	4.32 ± 0.64^Aa^	4.35 ± 0.37^Aa^	55.954	<0.05
Thearubigins	28.60 ± 2.12^Cc^	33.19 ± 2.14^Aa^	31.43 ± 1.57^Bb^	25.130	<0.05
Theabrownins	30.87 ± 3.54^Bb^	35.80 ± 3.11^Aa^	34.19 ± 3.38^Aa^	10.173	<0.05
TF	0.79 ± 0.14^Bb^	1.11 ± 0.18^Aa^	1.15 ± 0.20^Aa^	22.434	<0.05
TF-3-G	1.16 ± 0.18^Cc^	1.49 ± 0.26^Bb^	1.78 ± 0.23^Aa^	34.111	<0.05
TF-3′-G	0.36 ± 0.18^Ab^	0.44 ± 0.06^Aa^	0.46 ± 0.04^Aa^	4.279	<0.05
TFDG	0.29 ± 0.15^Bc^	1.02 ± 0.29^Aa^	0.81 ± 0.30^Ab^	38.534	<0.05
C	3.94 ± 0.96^Bb^	4.82 ± 0.60^Aa^	3.93 ± 0.92^Bb^	6.657	<0.05
EC	12.63 ± 1.66^Bb^	16.46 ± 1.68^Aa^	11.64 ± 2.12^Bb^	34.771	<0.05
EGC	28.97 ± 4.76^Bb^	36.30 ± 6.26^Aa^	23.39 ± 3.66^Cc^	30.057	<0.05
GC	1.18 ± 0.86^Bc^	1.81 ± 0.86^Bb^	2.78 ± 0.86^Aa^	15.922	<0.05
ECG	22.70 ± 4.16^Bc^	29.69 ± 3.66^Aa^	26.03 ± 3.54^Bb^	15.255	<0.05
GCG	4.22 ± 0.42^Bb^	4.95 ± 0.50^Aa^	3.46 ± 0.74^Cc^	30.720	<0.05
EGCG	51.51 ± 10.10^Aa^	55.60 ± 6.25^Aa^	50.44 ± 5.33^Aa^	2.364	≥0.05
CG	0.65 ± 0.11^Bb^	0.70 ± 0.09^Bb^	1.20 ± 0.30^Aa^	44.631	<0.05
Gallic acid	3.03 ± 0.95^Cc^	4.14 ± 0.80^Bb^	6.07 ± 0.66^Aa^	64.929	<0.05
Ellagic acid	2.85 ± 0.86^Aa^	2.58 ± 0.52^Aab^	2.25 ± 0.78^Ab^	3.009	≥0.05
TGG	2.06 ± 0.26^Aa^	1.91 ± 0.24^Aa^	2.03 ± 0.23^Aa^	1.973	≥0.05
Caffeine	37.48 ± 3.34^Bb^	42.97 ± 1.92^Aa^	41.36 ± 1.90^Ab^	23.335	<0.05
Theobromine	1.35 ± 0.20^Bb^	1.79 ± 0.61^Aa^	1.47 ± 0.21^ABa^	6.081	<0.05
Theophylline	0.264 ± 0.035^Aa^	0.222 ± 0.020^Bb^	0.252 ± 0.030^Aa^	9.950	<0.05
Rutin	1.57 ± 0.82^Bc^	3.18 ± 0.69^Aa^	1.91 ± 0.80^Bb^	21.662	<0.05
Taxifolin	0.61 ± 0.23^Bc^	0.93 ± 0.18^Aa^	0.79 ± 0.14^Ab^	12.911	<0.05
Myricetin	0.62 ± 0.26^Bb^	0.85 ± 0.24^Aa^	0.63 ± 0.24^ABb^	4.987	<0.05
Quercetin	0.105 ± 0.106^Ab^	0.124 ± 0.109^Ab^	0.196 ± 0.092^Aa^	3.917	<0.05
Luteolin	0.152 ± 0.100^Ab^	0.212 ± 0.085^Aa^	0.148 ± 0.062^Ab^	3.304	<0.05
Kaempferol	0.056 ± 0.053^Bb^	0.114 ± 0.043^Aa^	0.118 ± 0.070^Aa^	6.830	<0.05
l-theanine	13.33 ± 1.03^Ab^	9.85 ± 1.11^Bc^	14.25 ± 1.39^Aa^	68.531	<0.05
l-arginine	3.90 ± 0.93^Bb^	2.63 ± 0.48^Cc^	5.02 ± 0.65^Aa^	50.693	<0.05
l-aspartic acid	2.97 ± 0.30^Bb^	2.80 ± 0.35^Bb^	3.50 ± 0.34^Aa^	22.097	<0.05
l-asparagine	1.02 ± 0.16^Aa^	0.72 ± 0.13^Bb^	1.03 ± 0.14^Aa^	26.712	<0.05
l-histidine	0.89 ± 0.11^Aa^	0.61 ± 0.07^Bc^	0.81 ± 0.08^Ab^	48.869	<0.05
l-isoleucine	0.73 ± 0.07^Aa^	0.49 ± 0.09^Cc^	0.63 ± 0.07^Bb^	43.739	<0.05
l-glutamic acid	0.72 ± 0.10^Bb^	0.77 ± 0.08^Bb^	0.87 ± 0.08^Aa^	13.012	<0.05
l-phenylalanine	0.62 ± 0.11^Aa^	0.57 ± 0.06^Aa^	0.60 ± 0.07^Aa^	1.713	≥0.05
l-tyrosine	0.57 ± 0.04^Bb^	0.51 ± 0.03^Cc^	0.61 ± 0.06^Aa^	24.734	<0.05
l-alanine	0.53 ± 0.08^Bb^	0.44 ± 0.06^Cc^	0.60 ± 0.09^Aa^	21.799	<0.05
l-proline	0.56 ± 0.07^Aa^	0.49 ± 0.05^Bb^	0.58 ± 0.08^Aa^	8.666	<0.05
l-methionine	0.49 ± 0.04^Aa^	0.51 ± 0.05^Aa^	0.51 ± 0.04^Aa^	2.370	≥0.05
l-cysteine	0.37 ± 0.03^Ab^	0.31 ± 0.04^Bb^	0.40 ± 0.06^Aa^	22.324	<0.05
l-glycine	0.33 ± 0.04^Aa^	0.25 ± 0.03^Bb^	0.30 ± 0.03^Ab^	23.179	<0.05
l-leucine	0.32 ± 0.06^Aa^	0.26 ± 0.04^Bb^	0.32 ± 0.04^Aa^	10.447	<0.05
l-serine	0.30 ± 0.07^Aa^	0.29 ± 0.02^Aa^	0.30 ± 0.02^Aa^	4.316	≥0.05
l-valine	0.28 ± 0.02^ABab^	0.26 ± 0.03^Bb^	0.31 ± 0.07^Aa^	6.743	<0.05
l-threonine	0.27 ± 0.03^Bb^	0.22 ± 0.02^Cc^	0.30 ± 0.03^Aa^	39.688	<0.05
l-tryptophan	0.21 ± 0.03^Ab^	0.20 ± 0.03^Ab^	0.23 ± 0.05^Aa^	4.316	<0.05
GABA	0.081 ± 0.022^Bb^	0.107 ± 0.023^Aa^	0.104 ± 0.023^Aa^	6.952	<0.05

TF, theaflavin; TF-3-G, theaflavin-3-gallate; TF-3′-G, theaflavin-3′-gallate; TFDG, theaflavine-3,3′-digallate; C, (+)-catechin; EC, (−)-epicatechin; EGC, (−)-epigallocatechin; GC, (+)-gallocatechin; ECG, (−)-epicatechin gallate; GCG, (−)-gallocatechin gallate; EGCG, (−)-epigallocatechin gallate; CG, (−)-catechin gallate; TGG, 1,4,6-tri-*O-*galloyl-β-d-glucose. Total catechins were the summation of C, EC, EGC, GC, ECG, EGCG, GCG and CG contents. GABA, γ-aminobutyric acid.

Each tea sample was determined with three replications. Different uppercase and lowercase letters in superscript (A, B and C, *P* < 0.01; a, b and c, *P* < 0.05) in a same row indicate levels of statically significant difference determined by one-way ANOVA using Duncan‘s multiple range test. *F*-value and *P*-value were determined by ANOVA.

These chemical components could be assigned into 3 clusters (Fig. 3d), indicating the chemical distribution difference in white tea among Xinyang, Fuding and Yunnan Regions. For instances, Yunnan white tea contained relatively higher contents of components in cluster I, including seven catechins (i.e. C, EC, GC, EGC, ECG, GCG and EGCG), five flavonoids (i.e. rutin, myricetin, luteolin, kaempferol and taxifolin), four theaflavins (i.e. theaflavin, theaflavin-3-gallate, theaflavin-3′-gallate and theaflavine-3,3′-digallate), two tea pigments (i.e. thearubigins and theabrownins), two purine alkaloids (i.e. caffeine and theobromine) and gallic acid than other two groups, but relatively lower levels of components in cluster II, such as soluble sugars, GC, quercetin and eleven amino acids (i.e. l-proline, l-leucine, l-asparagine, l-theanine, l-valine, l-cysteine, l-tryptophan, l-alanine, l-threonine, l-arginine and l-aspartic acid). Generally, several amino acids and phenolic acid in cluster III kept relatively stable among three various regions, such as ellagic acid, l-methionine, l-tryptophan and l-glutamic acid, while Xinyang white tea had relatively higher l-serine, l-phenylalanine, l-histidine, l-isoleudine, l-glycine and theophylline.

### Comparison of seven quality components and four theaflavins among three production regions

Catechins content (a subgroup of flavanols) reached a level about 60–70% of tea polyphenols in the fresh tea-leaves (Fang et al., 2021), and varied significantly in tea processing (Zhou et al., 2022, Ma et al., 2021). Generally, catechins were decreased, and converted into theaflavins, thearubigins and theabrownins through oxidation reaction during the prolonged withering and aging of white tea, while several flavonoids were formed through the hydrolysis reaction of relevant flavonoid glycosides (Zhao et al., 2022, Zhou et al., 2022). The one-way ANOVA (Table 1) and independent-samples *t*-test (Fig. S1) demonstrated the significant (*P* < 0.05) differences of seven quality components and four theaflavins in the white tea among three production regions, which mainly attributed to tea tree variety or processing technology. The most abundance phenolic compounds of the fresh tea-leaves (*Camellia sinensis* var. *assamica*) guaranteed the highly significantly (*P* < 0.01) retention of tea polyphenols, total flavonoids and total catechins in Yunnan white tea after the prolonged withering and drying, while the long-term prolonged withering promoted the massive accumulation of thearubigins, theabrownins and theaflavine-3,3′-digallate in Fuding white tea. Additionally, the Fuding white tea still contained the highest contents of free amino acids and soluble sugars, which was consistent with the previous studies (Horanni and Engelhardt, 2013, Zhou et al., 2022). Despite the nuanced difference in tea cultivars and processing technology parameters, there were no highly significant (*P* ≥ 0.01) differences in tea polyphenols, total flavonoids and total catechins between Fuding and Xinyang white tea.

### Comparison of twenty bioactive compounds in white tea among three production regions

Except for EGCG, ellagic acid, quercetin, luteolin and TGG maintaining stability at *P* ≥ 0.05 or *P* ≥ 0.01 levels, the highly significantly (*P* < 0.01) differences of seven catechins, four flavonoids, three purine alkaloids and gallic acid were found in white teas among three production regions (Table 1 and Fig. S2). Through comparisons, the Yunnan white tea was observed to possess relatively higher levels of six catechins (i.e. C, EC, EGC, ECG, GCG and EGCG), four flavonoids (i.e. rutin, taxifolin, myricetin and luteolin) and two purine alkaloids (i.e. caffeine and theobromine), particularly rutin with a content of 3.18 mg/g that was 1.66 and 2.03 times of Xinyang and Fuding white tea. Conversely, Fuding white tea had highest levels of gallic acid, GC, CG, quercetin and kaempferol, which mainly came from the hydrolyzation of ester catechins like EGCG, and flavonoid glucosides such as rutin and astragalin (kaempferol-3-glucoside). Additionally, the relatively higher ellagic acid, TGG and theophylline contents in Xinyang white tea should be attributed to the regional climate and circumpolar latitude. Generally, the fresh tea-leaves (*Camellia sinensis* var. *assamica*) in Yunnan region generated the significantly (*P* < 0.01) higher contents of six catechins, four flavonoids and two purine alkaloids in Yunnan white tea.

The independent-samples *t*-test (Fig. S2) indicated the significantly (*P* < 0.05) differences of nine phenolic compounds including five catechins (i.e. EGC, GC, ECG, GCG and CG), two phenolic acids (i.e. gallic acid and ellagic acid) and two flavonoids (i.e. taxifolin and quercetin), as well as caffeine between Xinyang and Fuding white teas. Particularly, the long-term prolonged withering might lead to the relatively or significantly (*P* < 0.05) lower levels of non-ester catechins (i.e. EC and EGC), and significant (*P* < 0.05) higher contents of the three theaflavins (i.e. theaflavin, theaflavin-3-gallate and theaflavine-3,3′-digallate) and gallic acid in Fuding white tea, which promoted the mellow degree and reduced the astringency taste along with the enzymatic fermentation (Tan et al., 2017, Fan et al., 2021).

### Comparison of nineteen amino acids and GABA in white tea among three production regions

Through one-way ANOVA (Table 1), heat map analysis and independent-samples *t*-test (Fig. S3), the Fuding white tea has been confirmed to possess the highest free amino acids content, in particular twelve amino acids including l-theanine, l-arginine, l-aspartic acid, l-asparagine, l-glutamic acid, l-tyrosine, l-alanine, l-proline, l-cysteine, l-valine, l-threonine and l-tryptophan, which ensure the relatively higher umami degree (Fig. 1). The Yunnan large leaf species (*Camellia sinensis* var. *assamica*) such as Jinggu Dabaicha and Mengku Dayezhong cultivars, generated the lowest levels of amino acids in Yunnan white tea, particularly the major amino acids in tea-leaves, such as l-theanine, l-arginine and l-aspartic acid. Conversely, the Xinyang group cultivar, the special micro climatic environment in Xinyang region or the short-term prolonged withering might improve the contents of l-phenylalanine, l-histidine, l-isoleucine and l-glycine in Xinyang white tea.

### Comparison of the *in vitro* antioxidant activities among three production regions

The Yunnan white tea had significantly (*P* < 0.01) higher *in vitro* antioxidant capacity than the Xinyang or Fuding white teas, including FRAP, DPPH, ABTS, HSA and SSA, respectively (Table 2 and Table S8). Conversely, the Fuding white tea demonstrated the significantly (*P* < 0.05) lower *in vitro* antioxidant activities evaluated by DPPH and ABTS, but relatively higher FRAP than Xinyang white tea, because of its higher fermentation degree that caused significant (*P* < 0.05) reductions of several catechins, such as EGC, EC and GCG. However, no significant (*P* > 0.05) differences of DPPH and SSA were found between Xinyang and Fuding white teas. Generally, the significant (*P* < 0.05) higher ABTS and HSA in Xinyang white tea might be attributable to its relatively higher catechins level than Fuding white tea, such as EGC, EC, EGCG and GCG.

**Table 2:** The *in vitro* antioxidant activities of FRAP, DPPH, ABTS, HSA and SSA, and their differences among Xinyang, Fuding and Yunnan Regions.

Antioxidant ability assay	Production regions			ANOVA	
	Xinyang (n = 6 × 3)	Yunan (n = 6 × 3)	Fuding (n = 6 × 3)	*F*-value	P-value
FARP (μmol trolox/g)	1693.93 ± 78.97^Bb^	1767.55 ± 44.20^Aa^	1710.17 ± 56.78^Bb^	7.08	<0.05
DPPH (mg trolox/g)	555.64 ± 22.39^Bb^	594.22 ± 11.02^Aa^	563.95 ± 20.49^Bb^	21.36	<0.05
ABTS (mg trolox/g)	304.02 ± 29.38^Bb^	330.27 ± 24.47^Aa^	279.18 ± 33.56^Bc^	13.62	<0.05
HSA (%)	67.23 ± 5.26^Bb^	72.14 ± 2.73^Aa^	63.55 ± 4.83^Bc^	17.16	<0.05
SSA (%)	39.32 ± 2.87^Bb^	43.22 ± 2.14^Aa^	37.74 ± 2.64^Bb^	21.68	<0.05

FRAP: Ferric ion reducing antioxidant power; DPPH, DPPH free radical scavenging activity; ABTS, ABTS^•+^ scavenging activity; HSA, hydroxyl radical scavenging ability; SSA, superoxide anion radical scavenging ability. Each tea sample was determined with three replications. Different uppercase and lowercase letters in superscript (A, B and C, *P* < 0.01; a, b and c, *P* < 0.05) in a same row indicate levels of statically significant difference determined by one-way ANOVA using Duncan‘s multiple range test. *F*-value and *P*-value were determined by ANOVA.

### Chemicals associated with tastes and antioxidant activities in Chinese white teas

**Fig. 4:**
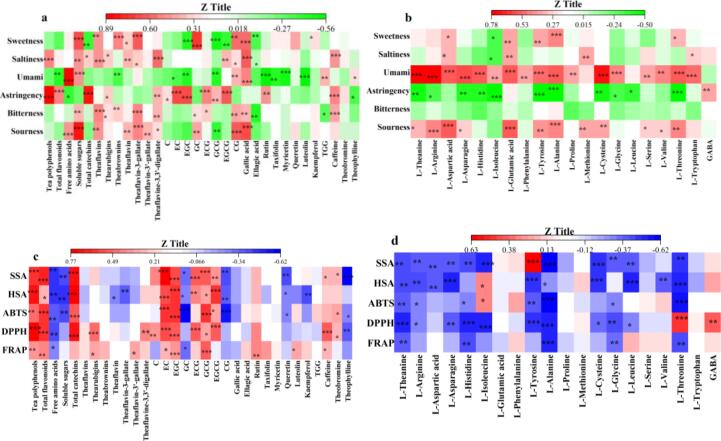
Impacts of quality components and bioactive compounds (a, c), and amino acids and GABA (b, d) on tastes (a, b) and *in vitro* antioxidant activities (c, d) in white teas through the bivariate correlation analysis.

ECG, EGCG, kaempferol/quercetin glycosides, caffeine and non-ester catechins were main contributors correlated to astringent and bitterness intensity in tea-leaves (Jiang et al., 2022, Zhang et al., 2020), in which most of them have been regarded as the major antioxidants in tea, such as EGCG and ECG (Tang et al., 2019). Amino acids conduced to the umami taste (Hajeb and Jinap, 2015, Hayashi et al., 2008), while GABA, rutin and gallic acid negatively impacted the sweetness taste, and had potential correlations with the antioxidant activities (Li et al., 2019, Yu and Yang, 2019). Combined with the E-tongue results, the bivariate correlation analysis (Fig. 4a, Fig. 4b and Table S9) revealed the significant correlations of the chemical components such as phenolic compounds, purine alkaloids and amino acids, to tastes factors including sourness, astringency and umami with a relatively higher Person correlation coefficient (r-value > 0.50 or r < -0.5, and *P* < 0.001). Concretely, tea polyphenols, total flavonoids, four catechins (i.e. EC, EGC, ECG and EGCG), and caffeine showed the significantly (*P* < 0.001) positive (r > 0.50) correlations to astringency, and their hydrolyzation, oxidation and condensation during the prolonged withering reduced the astringent intensity in white tea. Due to the significantly (*P* < 0.001) negative correlations of several amino acids such as l-isoleucine, l-tyrosine, l-alanine and l-threonine to astringency, the relatively higher contents of these specific amino acids might further reduce the astringent intensity in Xinyang white tea to a certain degree. As the potential umami substances for the significantly (*P* < 0.001) positive (r > 0.50) correlations, the ten amino acids (i.e. l-theanine, l-arginine, l-aspartic acid, l-asparagine, l-histidine, l-glutamic acid, l-tyrosine, l-alanine, l-threonine, and l-cysteine) improved the umami intensity in white tea. Additionally, except for several amino acids, such as l-glutamic acid, l-aspartic acid, l-arginine and l-alanine, gallic acid, theaflavin-3-gallate and CG also demonstrated the significantly (*P* < 0.001) positive (r > 0.50) correlations with the sourness in white tea, which might lead to the significant higher sourness value in Fuding white tea.

As the major bitter compound, caffeine showed a positive correlation to the bitterness in white tea, which was consistent with the previous study (Suess, Brockhoff, Meyerhof, & Hofmann, 2018). Additionally, the significantly (*P* < 0.001) positive (r > 0.50) correlations of the theaflavins (r = 0.502) particularly theaflavin-3-gallate (r = 0.517) to the bitterness, indicated that the slight enzymatic oxidation with the accumulation of theaflavins might enhance the bitterness in white tea (Zhou, Ma et al., 2022). Conversely, soluble sugars, theabrownins, theaflavin-3-gallate, CG and gallic acid demonstrated the significantly (*P* < 0.001) positive (r > 0.50) correlations to the sweetness taste in white tea. Furthermore, those chemical bio-transformations might also influence the aroma and taste of white tea during the storage (Fan et al., 2021; S. Xu et al., 2019).

As shown in Fig. 4c, Fig. 4d and Table S10, the catechins such as EC, EGC, ECG, GCG and EGCG, two purine alkaloids (i.e. caffeine and theophylline), and several amino acids including l-theanine, l-histidine, l-isoleucine, l-alanine, l-tyrosine, l-leucine and l-threonine showed the significantly (*P* < 0.001) positive (r > 0.50) or negative (r < -0.5) correlations to the *in vitro* antioxidant abilities. Particularly, the five catechins such as EC, EGC, ECG, GCG and EGCG could be regarded as the main antioxidants in Chinese white tea. However, the relatively lower extraction rate of flavonoids in the antioxidant capacity test limited the exploration about the internal connections between flavonoids and antioxidant activities in white teas. Several amino acids could reduce the antioxidant activities, because of the formation of oligomer together with relevant phenolic compounds.

## Conclusions

Based on E-tongue results, and fifty-one chemical components contents determined by HPLC and amino acid analyzer, the multivariate statistical analyses, such as PCA, HCA and PLS-DA completely divided these white teas into Xinyang, Fuding and Yunnan white tea groups in accordance with its origins, which indicated the feasibility of Chinese white teas classification by production regions at a national scale. Twelve characteristic compounds (VIP > 1.0 and *P* < 0.05) were selected for the geographical identification of Chinese white teas. The contrastive analysis such as one-way ANOVA and the independent-samples *t*-test revealed their specific differences of sensory tastes, chemical components and *in vitro* antioxidant ability in white tea among three production regions (i.e. Xinyang, Fuding and Yunnan), which mainly attributed to the locally grown varieties/cultivars, processing parameters and other environmental factors, such as regional climate and unique geographical conditions. The bivariate correlation analysis indicated that EC, EGC, ECG, EGCG and caffeine enhanced the bitter and astringent taste, while the ten amino acids were the main umami substances in white tea. Additionally, the five catechins promoted the antioxidant capacity in white tea. In conclusion, this study elaborating the classification of Chinese white tea by production region, and their specific differences in tastes, chemical components and antioxidant capacity among three main production regions, will contribute to the geographical identification of white teas.

## Ethical statement

Ethics approval was not required for this research that did not involve any human or animal testing.

## CRediT authorship contribution statement

**Cunqiang Ma:** Methodology, Data curation, Visualization, Software, Writing – original draft, Writing – review & editing, Project administration. **Bingsong Ma:** Investigation, Data curation, Formal analysis, Resources. **Jiacai Wang:** Investigation, Methodology, Data curation. **Zihao Wang:** Conceptualization, Investigation, Funding acquisition. **Xuan Chen:** Project administration, Conceptualization, Writing – review & editing, Funding acquisition. **Binxing Zhou:** Conceptualization, Investigation, Formal analysis, Validation, Writing – original draft, Funding acquisition. **Xinghui Li:** Project administration, Conceptualization, Writing – review & editing, Funding acquisition.

## Declaration of Competing Interest

The authors declare that they have no known competing financial interests or personal relationships that could have appeared to influence the work reported in this paper.

## Data Availability

Data will be made available on request.
