# Supplementary material for: Geographical origin identification of Chinese white teas, and their differences in tastes, chemical compositions and antioxidant activities among three production regions
Source: Food Chem X. 2022 Nov 7;16:100504. doi: 10.1016/j.fochx.2022.100504 (PMC9743341; doi:10.1016/j.fochx.2022.100504)
Supplement: Supplementary data 1 [file mmc1.doc]

**Table S1. Information of eighteen Bai mudan sub-type of spring white tea samples from three various origins.**

| No. | Production places | Cultivars | Size | Season |
| --- | --- | --- | --- | --- |
| XYT-1 | Shihe District Xinyang | Xinyang group | One bud two leaves | Spring |
| XYT-2 | Shihe District Xinyang | Xinyang group | One bud two leaves | Spring |
| XYT-3 | Shihe District Xinyang | Fuding Dabaicha | One bud two leaves | Spring |
| XYT-4 | Shihe District Xinyang | Fuding Dabaicha | One bud two leaves | Spring |
| XYT-5 | Shihe District Xinyang | Fuding Dabaicha | One bud two leaves in early development | Spring |
| XYT-6 | Shihe District Xinyang | Xinyang group | One bud two leaves | Spring |
| YNT-1 | Lincang of Yunnan | Mengku Dayezhong | One bud two leaves in early development | Spring |
| YNT-2 | Lincang of Yunnan | Mengku Dayezhong | One bud two leaves in early development | Spring |
| YNT-3 | Puer of Yunan | Jinggu Dabaicha | One bud two leaves | Spring |
| YNT-4 | Puer of Yunan | Jinggu Dabaicha | One bud two leaves | Spring |
| YNT-5 | Lincang of Yunnan | Mengku Dayezhong | One bud two leaves in early development | Spring |
| YNT-6 | Lincang of Yunnan | Mengku Dayezhong | One bud two leaves in early development | Spring |
| FDT-1 | Fuding of Fujian | Fuding Dabaicha | One bud two leaves | Spring |
| FDT-2 | Fuding of Fujian | Fuding Dabaicha | One bud two leaves | Spring |
| FDT-3 | Fuding of Fujian | Fuding Dahaocha | One bud two leaves | Spring |
| FDT-4 | Fuding of Fujian | Fuding Dahaocha | One bud two leaves in early development | Spring |
| FDT-5 | Fuding of Fujian | Fuding Dahaocha | One bud two leaves | Spring |
| FDT-6 | Fuding of Fujian | Fuding Dahaocha | One bud two leaves | Spring |

**Table S2.** HPLC method validation for the determination of seventeen phenolic compounds and three purine alkaloids.

| Compounds | Retention time (min) | Equations of standard curve | R^2^ |
| --- | --- | --- | --- |
| Gallic acid | 4.039 | y = 14.816x - 73.836 | 0.9991 |
| (+)-Gallocatechin (GC) | 5.803 | y= 0.6627x -3.9576 | 0.9995 |
| Theabromine | 6.259 | y = 6.9893x + 1.1963 | 0.9947 |
| Theaphylline | 8.817 | y = 7.7339x - 51.562 | 0.9995 |
| (-)-Epigallocatechin (EGC) | 9.757 | y = 0.4872x + 5.5409 | 0.9904 |
| (+)-Catechin (C) | 9.978 | y = 1.3724x + 16.687 | 0.9907 |
| Caffeine | 11.360 | y = 7.852x - 37.104 | 0.9967 |
| (-)-Epigallocatechin gallate (EGCG) | 12.533 | y = 7.1084x -190.28 | 0.9955 |
| (-)-Epicatechin (EC) | 13.677 | y = 3.8417x - 43.483 | 0.9962 |
| (-)-Gallocatechin gallate (GCG) | 14.489 | y = 4.322x - 68.856 | 0.9947 |
| 1,4,6-Tri-*O-*galloyl-β-D-glucose (GG) | 15.784 | y = 3.8325x + 10.754 | 0.9976 |
| (-)-Epicatechin gallate (ECG) | 16.367 | Y = 4.865x - 0.271 | 0.9911 |
| Taxifolin | 17.162 | y = 5.7379x - 40.887 | 0.9996 |
| (-)-Catechin gallate (CG) | 17.623 | y = 4.3366x - 31.784 | 0.9995 |
| Rutin | 21.051 | y = 4.3966x - 15.192 | 0.9913 |
| Ellagic acid | 21.936 | y = 4.0000x - 35.983 | 0.9981 |
| Myricetin | 22.483 | y = 11.042x - 60.697 | 0.9989 |
| Quercetin | 24.998 | y = 9.3203x - 63.393 | 0.9994 |
| Luteolin | 25.593 | y = 11.139x - 70.867 | 0.9995 |
| Kaempferol | 26.349 | y = 9.716x - 63.798 | 0.9995 |

Note: y, peak area (mAU*s), x, concentration of analyte (μg/mL).

**Table S3. HPLC method validation for the determination of four theaflavins.**

| Compounds | Retention time (min) | Equations of standard curve | R^2^ |
| --- | --- | --- | --- |
| Theaflavin | 25.580 | y = 6.8248x - 66.396 | 0.9994 |
| Theaflavin-3-gallate | 26.581 | y = 8.2251x - 34.356 | 0.9990 |
| Theaflavin-3'-gallate | 27.296 | y = 8.4996x - 5.6029 | 0.9993 |
| Theaflavine-3,3'-digallate | 27.102 | y = 10.967x - 68.366 | 0.9991 |

Note: y, peak area (mAU*s), x, concentration of analyte (μg/mL).

**Table S4.** Method validation for the determination of nineteen amino acids and GABA by amino acid analyzer.

| Compounds | Retention time (min) | Equations of standard curve | R^2^ |
| --- | --- | --- | --- |
| L-aspartic acid | 11.425 | y = 9.788x + 1.4879 | 0.9930 |
| L-threonine | 16.305 | y = 9.2866 - 7.1048 | 0.9952 |
| L-serine | 17.963 | y = 8.5127x + 5.6447 | 0.9997 |
| L-glutamic acid | 22.827 | y = 4.3499x + 4.3999 | 0.9956 |
| L-theanine | 28.125 | y = 1.5197x - 9.4296 | 0.9965 |
| L-glycine | 36.428 | y = 8.9727x + 3.997 | 0.9983 |
| L-alanine | 37.738 | y = 1022x + 3.3742 | 0.9998 |
| L-valine | 42.838 | y =16.606x + 3.8102 | 0.9990 |
| L-cysteine | 43.755 | y = 15.169x - 7.7352 | 0.9903 |
| Lmethionine | 46.405 | y = 12.855x + 23.032 | 0.9996 |
| L-isoleucine | 50.214 | y = 15.838x - 15.627 | 0.9989 |
| L-leucine | 52.055 | y = 15.411x + 13.598 | 0.9988 |
| L-tyrosine | 53.592 | y = 7.5925x + 15.697 | 0.9966 |
| L-phenylalanine | 58.125 | y = 9.8581x + 10.582 | 0.9994 |
| γ-aminobutyric acid (GABA) | 66.402 | y = 17.37x + 53.178 | 0.9992 |
| L-tryptophan | 70.726 | y = 3.1278x - 5.664 | 0.9966 |
| L-histidine | 96.227 | y = 14.336x + 3.4353 | 0.9999 |
| L-arginine | 108.728 | y = 9.0849x - 4.2452 | 0.9991 |
| L-asparagine | 23.405 | y = 9.2488x + 4.1848 | 0.9948 |
| L-proline | 37.527 | y = 6.9514x + 8.4824 | 0.9980 |

Note: y, peak area (mAU*s), x, concentration of analyte (μg/mL).

**Table S5.** Description of traditional sensory evaluation of eighteen white tea samples.

| No. | Origins | Shape | Liquor color | Aroma | Taste | Tea-leaves residues |
| --- | --- | --- | --- | --- | --- | --- |
| XYT-1 | Xinyang | Neat tender leaves with jade green | Yellow cream,clear and bright | Pure and lasting fragrance with pekoe flavour | Fresh, mellow, sweet and thick | Tender fatness, evenly, bright yellow green |
| XYT-2 | Xinyang | Fat bud and unfolded leaves with glossy greyish-green | Yellow-green, clear and fresh | Fresh-sweet,pure and neutral | Heavy fresh, sweet and mellow | Tender fatness, still evenly, yellow-green |
| XYT-3 | Xinyang | Whole shoot, greyish-green leaf side roll down with white hair | Light yellow, clear and bright | Obvious fresh fruity | Mellow,no bitterness and astringent taste | Tender fatness with slightly mixed yellow-green |
| XYT-4 | Xinyang | Unfolded, greyish-green or jade green leaves,evenly | Yellow-green with a little precipitate | Pure and neutral fragrance | Mellow and neutral sweetness | Fresh and tender reside leaves with mixed yellow-green |
| XYT-5 | Xinyang | White and fat buds, green and even leaves | Bright yellow,clear liquor | Lofty fresh with slight pekoe flavour | Fresh and mellow | Tender and plump with yellow-green |
| XYT-6 | Xinyang | Silvery buds and greyish-green leaves,still evenly | Light yellow or green yellow, clear and bright | Pure and lasting fragrance, fresh sweet aroma | Sweet, fresh and mellow | Fresh, still evenly and slight mixed yellow-green |
| YNT-1 | Yunnan | Silvery buds,unfolded grey-white leaves | Green-yellow,clear and marginal bright | Fragrant aroma with fresh fruity | Full mellow and thick with slight astringent | Mixed tender fatness |
| YNT-2 | Yunnan | Fat buds, Whole shoot with grey-white color | apricot yellow,clear and marginal bright | Pure, fresh and sweet fragrant aroma | Sweet,mellow and thick, still fresh | Soft, flower-like, evenly |
| YNT-3 | Yunnan | Silvery fat buds, white and dark levels | Orange-yellow,clear and bright | Pure, lasting sweet aroma with slight mature fruity | Sweetness, mellow and thick | Soft and fatness, flower-like |
| YNT-4 | Yunnan | Pure white vellus in buds, and slivery white leaves | Orange-yellow,clear and marginal bright | Pure and mature fruity aroma | Sweetness, mellow and thick, no bitter/astringency | Fresh and tender with red leaf |
| YNT-5 | Yunnan | Whole shoots with greyish-green or jade-green leaves | Yellow-green, clear and bright | Lasting fragrant aroma with fresh sweetness | Mellow, thick and fresh | Mixed tender fatness |
| YNT-6 | Yunnan | Greyish-green silky leaves and silvery-white buds | Slight yellow or green yellow, clear and bright | Sweet and fragrant aroma | Heavy, thick, fresh, mellow | Fresh and iron greyish-green reside leaves |
| FDT-1 | Fuding | Whole shoots with fat buds and fresh greyish-green leaves | Apricot yellow,clear and bright | Flowers fragrant with pekoe flavour | Sweet, fresh, mellow and brisk | Detached buds and leaves with reddish veins |
| FDT-2 | Fuding | Whole shoots with greyish-green or jade green leaves and white hairs | Orange-yellow, clear and bright | Lasting flowery fragrant | Fresh, mellow and obvious sweet | Light grey, soft reside leaves with reddish veins |
| FDT-3 | Fuding | Dark gray-green leaves and white hairs | Orange-yellow, clear and bright | Obvious flowery and slight sweet aroma | Sweet, mellow, heavy and thick | Whole gray-green shoots with mixed color |
| FDT-4 | Fuding | Obvious white pekoe, and gray-green levels, partial reddish brown leaves | Apricot yellow, clear and bright | Obvious pekoe flavour and flowery | Fresh, mellow, sweet | Whole slight -gray buds and leaves, fresh and bright |
| FDT-5 | Fuding | Whole gray-green silky shoots with side roll down leaf | Orange-yellow, clear and bright | Slight pekoe flavour and delicate fragrance | Sweet, fresh and mellow | Fresh tender bright leaves with slight red leaf |
| FDT-6 | Fuding | Gray-green or iron grey leaves with white pekoe | Apricot yellow, clear and bright | Fresh and pure fragrant aroma | Fresh, sweet and mellow | Fat buds and bright slight gray leaves |

**Table S6.** Traditional sensory evaluation scores of eighteen white tea samples.

| No. | Shape | Liquor color | Aroma | Taste | Tea-leaves residues | Total score |
| --- | --- | --- | --- | --- | --- | --- |
| XYT-1 | 82.2±0.84 | 86.8±1.48 | 79.2±2.39 | 79.0±2.74 | 86.2±1.92 | 81.20±0.42 |
| XYT-2 | 78.4±2.79 | 84.4±1.82 | 83.2±1.30 | 80.0±1.58 | 86.8±1.48 | 81.76±0.52 |
| XYT-3 | 76.4±1.14 | 85.8±1.92 | 81.4±1.52 | 83.2±1.92 | 83.0±1.58 | 81.54±0.29 |
| XYT-4 | 79.2±2.39 | 83.0±2.55 | 81.8±1.30 | 81.2±3.83 | 82.2±1.92 | 81.26±1.40 |
| XYT-5 | 82.6±0.89 | 81.8±1.92 | 80.8±2.17 | 82.8±2.58 | 81.6±2.07 | 81.94±1.49 |
| XYT-6 | 84.0±1.58 | 79.8±2.59 | 81.4±1.52 | 83.4±1.14 | 83.4±1.14 | 82.56±0.78 |
| YNT-1 | 76.2±1.79 | 81.2±2.59 | 78.2±2.49 | 82.0±2.24 | 80.4±2.88 | 79.46±1.24 |
| YNT-2 | 73.6±1.34 | 82.6±2.30 | 81.4±1.52 | 78.6±3.51 | 84.6±1.14 | 79.44±0.60 |
| YNT-3 | 79.6±3.36 | 76.6±1.52 | 81.8±1.92 | 81.0±1.41 | 85.0±1.87 | 80.92±0.36 |
| YNT-4 | 76.8±1.64 | 80.6±2.07 | 76.0±2.74 | 83.8±1.48 | 76.0±2.24 | 78.96±0.71 |
| YNT-5 | 78.6±2.30 | 79.0±2.55 | 75.5±2.50 | 79.6±4.34 | 82.6±1.14 | 78.41±1.33 |
| YNT-6 | 78.4±2.79 | 78.6±2.79 | 79.2±1.30 | 83.4±1.67 | 83.2±1.48 | 80.64±0.52 |
| FDT-1 | 83.4±3.05 | 86.6±1.52 | 84.0±1.58 | 84.8±1.64 | 83.2±2.68 | 84.30±0.46 |
| FDT-2 | 84.6±1.14 | 86.4±1.67 | 83.3±2.82 | 84.4±2.07 | 82.4±2.41 | 84.11±1.40 |
| FDT-3 | 82.0±2.24 | 89.8±3.11 | 84.4±1.14 | 81.8±1.92 | 81.6±1.67 | 83.40±0.71 |
| FDT-4 | 84.6±1.14 | 82.8±0.84 | 84.0±2.12 | 83.2±1.30 | 82.6±2.30 | 83.62±0.71 |
| FDT-5 | 81.0±1.87 | 85.6±2.07 | 86.8±3.35 | 83.8±0.84 | 82.8±1.92 | 84.22±1.11 |
| FDT-6 | 81.4±2.51 | 85.6±2.41 | 82.8±2.59 | 84.6±1.14 | 82.2±2.17 | 83.28±0.45 |

Note: Each tea sample was determined with five replications.

Total score = 20% × appearance (a) + 10% × liquor color (b) + 30% × aroma (c) + 30% × taste (d) + 10% × tea-leaves residues (e).

**Table S7.** Difference of sensory evaluation results in white teas among three production regions, i.e., Xinyang, Yunnan and Fuding regions.

| Factors | Production regions | | | ANOVA | |
| --- | --- | --- | --- | --- | --- |
|  | Xinyang (n=6*3) | Yunnan (n=6*3) | Fuding (n=6*3) | F-value | P-value |
| Shape | 80.47±3.15^Bb^ | 77.20±2.91^Cc^ | 82.83±2.42^Aa^ | 29.729 | < 0.05 |
| Liquor color | 83.60±3.07^Bb^ | 79.77±2.91^Cc^ | 86.13±2.81^Aa^ | 35.849 | < 0.05 |
| Aroma | 81.30±2.00^Bb^ | 79.68±3.14^Cc^ | 84.22±2.52^Aa^ | 34.103 | < 0.05 |
| Taste | 81.60±2.81^Bb^ | 81.40±3.10^Bb^ | 83.77±1.76^Aa^ | 7.521 | < 0.05 |
| Tea-leaves residues | 83.87±2.53^Aa^ | 81.97±3.56^Ab^ | 82.47±2.08^Aab^ | 3.735 | < 0.05 |
| Total score | 81.71±0.98^Bb^ | 79.64±1.20^Cc^ | 83.82±0.87^Aa^ | 125.083 | < 0.05 |

Different uppercase and lowercase letters in superscript (A, B and C, *P* < 0.01; a, b and c, *P* < 0.05) in a same row indicate levels of compounds with statically significant difference determined by one-way ANOVA using Duncan`s multiple comparative analysis method. *F*-value and *P*-value were determined by ANOVA.

**Table S8.** *In vitro* antioxidant activities of FRAP, DPPH. ABTS, HSA and SSA in white tea collected from Xinyang, Yunnan and Fuding regions, respectively.

| Assays | Xinyang white tea (n=6*3) | | Yunan white tea (n=6*3) | | Fuding white tea (n=6*3) | |
| --- | --- | --- | --- | --- | --- | --- |
|  | Content | Rang | Content | Rang | Content | Rang |
| FRAP (μmol Trolox/g) | 1693.93±78.97^Bb^ | 1654.66-1733.21 | 1767.55±44.20^Aa^ | 1745.57-1789.53 | 1710.17±56.78^Bb^ | 1681.93-1738.40 |
| DPPH (mg Trolox/g) | 555.64±22.39^Bb^ | 544.51-566.77 | 594.22±11.02^Aa^ | 588.74-599.71 | 563.95±20.49^Bb^ | 553.76-574.14 |
| ABTS (mg Trolox/g) | 304.02±29.38^Bb^ | 289.41-318.63 | 330.27±24.47^Aa^ | 318.09-342.44 | 279.18±33.56^Bc^ | 262.49-295.87 |
| HSA (%) | 67.23±5.26^Bb^ | 64.61-69.84 | 72.14±2.73^Aa^ | 70.78-73.50 | 63.54±4.83^Bc^ | 61.14-65.95 |
| SSA (%) | 39.32±2.87^Bb^ | 37.90-40.75 | 43.22±2.14^Aa^ | 42.15-44.28 | 37.74±2.64^Bb^ | 136.43-39.06 |

FRAP: Ferric ion reducing antioxidant power; DPPH, DPPH free radical scavenging activity; ABTS, ABTS•+ scavenging activity; HSA, hydroxyl radical scavenging ability; SSA, superoxide anion radical scavenging ability.

Each tea sample was determined with three replications. Different uppercase and lowercase letters in superscript (A, B and C, *P*<0.01; a, b and c, *P*<0.05) in a same row indicate levels of compounds with statically significant difference determined by one-way ANOVA using Duncan`s multiple comparative analysis method.

**Table S9. Correlations of chemical components including phenolic compounds, theaflavins, purine alkaloids, amino acid and GABA to six tastes determined by E-tongue.**

| Components | Sourness | Bitterness | Astringency | Umami | Saltiness | Sweetness |
| --- | --- | --- | --- | --- | --- | --- |
| Tea polyphenols | -0.016 | -0.087 | 0.883*** | -0.234 | 0.510*** | -0.144 |
| Total flavonoids | -0.150 | 0.153 | 0.520*** | -0.436** | 0.226 | -0.296* |
| Free amino acids | 0.488*** | -0.009 | -0.374* | 0.875*** | 0.148 | 0.257 |
| Soluble sugars | 0.798*** | 0.385** | -0.235 | 0.462*** | 0.452** | 0.669*** |
| Total catechins | -0.183 | -0.008 | 0.821*** | -0.205 | 0.345* | -0.381** |
| Theaflavins | 0.457** | 0.502*** | 0.113 | -0.100 | 0.488*** | 0.455** |
| Thearubigins | 0.251 | 0.298* | 0.336* | -0215 | 0.33* | 0.198 |
| Theabrownins | 0.184 | 0.349** | -0.043 | -0.356** | 0.032 | 0.511*** |
| Theaflavin | 0.393** | 0.228 | 0.066 | -0.052 | 0.367** | 0.343* |
| Theaflavin-3-gallate | 0.537*** | 0.517*** | -0.134 | 0.212 | 0.324* | 0.628*** |
| Theaflavin-3'-gallate | 0.376** | 0.195 | -0.088 | -0.052 | 0.244 | 0.199 |
| Theaflavine-3,3'-digallate | 0.298* | 0.369** | 0.387** | -0.219 | 0.572*** | 0.247 |
| C | -0.171 | 0.003 | 0.306* | -0.264 | 0.004 | -0.089 |
| EC | -0.236 | -0.137 | 0.646*** | -0.348* | 0.136 | -0.215 |
| EGC | -0.334* | -0.101 | 0.655*** | -0.430** | 0.174 | -0.460*** |
| GC | 0.412** | 0.302* | -0.224 | 0.057 | 0.179 | 0.696*** |
| ECG | 0.091 | 0.318* | 0.524*** | -0.001 | 0.252 | 0.020 |
| GCG | -0.414** | -0.068 | 0.358** | -0.452** | -0.092 | -0.487*** |
| EGCG | -0.080 | -0.121 | 0.625*** | 0.133 | 0.374** | -0.406** |
| CG | 0.600*** | 0.421** | -0.221 | 0.357** | 0.298* | 0.559*** |
| Gallic acid | 0.657*** | 0.390** | 0.063 | 0.527*** | 0.601*** | 0.587*** |
| Ellagic acid | -0.233 | -0.441** | 0.049 | -0.299* | -0.051 | -0.366** |
| Rutin | -0.117 | 0.116 | 0.463** | -0.541*** | 0.253 | -0.019 |
| Taxifolin | 0.177 | 0.190 | 0.011 | -0.424** | 0.181 | 0.248 |
| Myricetin | 0.003 | -0.026 | 0.074 | -0.478*** | 0.084 | 0.214 |
| Quercetin | 0.036 | 0.055 | 0.410 | -0.156 | 0.240 | 0.209 |
| Luteolin | -0.117 | 0.181 | -0.199 | -0.556*** | -0.138 | 0.017 |
| Kaempferol | 0.352 | 0.237 | -0.057 | 0.066 | 0.072 | 0.218* |
| TGG | 0.136 | -0.290* | 0.084 | 0.380** | 0.184 | -0.047 |
| Caffeine | 0.301 | 0.433** | 0.502*** | 0.042 | 0.468*** | 0.118 |
| Theobromine | 0.011 | 0.041 | 0.260 | -0.120 | 0.221 | -0.072 |
| Theophylline | -0.006 | -0.181 | -0.341* | 0.290* | -0.029 | 0.128 |
| L-theanine | 0.344* | -0.088 | -0.435** | 0.776*** | -0.004 | 0.193 |
| L-arginine | 0.477*** | 0.037 | -0.295* | 0.779*** | 0.197 | 0.228 |
| L-aspartic acid | 0.561*** | -0.036 | -0.108 | 0.526*** | 0.350* | 0.333* |
| L-asparagine | 0.304* | -0.191 | -0.410** | 0.589*** | -0.047 | 0.061 |
| L-histidine | 0.028 | -0.247 | -0.410** | 0.536*** | 0.210 | -0.057 |
| L-isoleucine | -0.173 | -0.113 | -0.475*** | 0.368** | -0.292* | -0.301* |
| L-glutamic acid | 0.526*** | 0.057 | 0.198 | 0.552*** | 0.387** | 0.371** |
| L-phenylalanine | 0.047 | -0.041 | 0.006 | 0.375** | -0.020 | -0.142 |
| L-tyrosine | 0.415** | 0.146 | -0.493*** | 0.519*** | 0.090 | 0.390** |
| L-alanine | 0.474*** | 0.198 | -0.475*** | 0.547*** | 0.040 | 0.461*** |
| L-proline | 0.165 | -0.244 | -0.095 | 0.387** | 0.147 | 0.097 |
| L-methionine | 0.379** | -0.178 | 0.112 | 0.265 | 0.378** | 0.217 |
| L-cysteine | 0.377** | 0.136 | -0.441** | 0.707*** | 0.103 | 0.123 |
| L-glycine | 0.091 | -0.180 | -0.317* | 0.480*** | -0.176 | -0.134 |
| L-leucine | 0.102 | -0.093 | -0.338* | 0.201 | -0.029 | 0.045 |
| L-serine | 0.310* | -0.061 | 0 | 0.390** | 0.243 | -0.163 |
| L-valine | 0.303* | -0.061 | -0.128 | 0.456** | 0.042 | 0.043 |
| L-threonine | 0.450** | -0.017 | -0.470*** | 0.553*** | -0.069 | 0.342* |
| L-tryptophan | 0.252 | -0.174 | 0.055 | 0.483*** | 0.292* | 0.093 |
| GABA | 0.083 | 0.168 | 0.374** | -0.054 | 0.241 | 0.132 |

Person correlation coefficient was determined by the bivariate correlation analysis using SPSS 20.0 software.

Significant levels: *** indicated *P* < 0.001, ** indicated 0.001 ≤ *P* < 0.01, *indicated 0.01 ≤ *P* < 0.05.

**Table S10. Correlations of chemical components including phenolic compounds, theaflavins, purine alkaloids, amino acid and GABA to *in vitro* antioxidant abilities evaluated by five various assays.**

| Components | FRAP | DPPH | ABTS | HSA | SSA |
| --- | --- | --- | --- | --- | --- |
| Tea polyphenols | 0.355** | 0.734*** | 0.487*** | 0.688*** | 0.615*** |
| Total flavonoids | 0.434** | 0.623*** | 0.599*** | 0.291* | 0.638*** |
| Free amino acids | -0.288* | -0.402** | -0.351** | -0.455** | -0.447** |
| Soluble sugars | 0.002 | -0.180 | -0.395** | -0.443** | -0.417** |
| Total catechins | 0.268 | 0.710*** | 0.532*** | 0.744*** | 0.673*** |
| Theaflavins | 0.176 | 0.208 | -0.091 | -0.131 | -0.052 |
| Thearubigins | 0.335* | 0.482*** | 0.11 | 0.049 | 0.237 |
| Theabrownins | 0.266 | 0.061 | 0.027 | -0.058 | -0.081 |
| Theaflavin | 0.107 | 0.158 | -0.184 | -0.284* | -0.078 |
| Theaflavin-3-gallate | 0.048 | -0.037 | -0.202 | -0.376** | -0.181 |
| Theaflavin-3'-gallate | 0.314* | 0.032 | 0.076 | -0.178 | -0.124 |
| Theaflavine-3,3'-digallate | 0.219 | 0.423** | 0.082 | 0.225 | 0.107 |
| C | -0.197 | 0.373** | 0.017 | 0.225 | 0.408** |
| EC | 0.301* | 0.540*** | 0.605*** | 0.579*** | 0.740*** |
| EGC | 0.388** | 0.511** | 0.597*** | 0.617*** | 0.547*** |
| GC | -0.329* | -0.141 | -0.616*** | -0.283 | -0.335* |
| ECG | 0.223 | 0.503*** | 0.181 | 0.417** | 0.506*** |
| GCG | 0.499*** | 0.323* | 0.611*** | 0.313* | 0.524*** |
| EGCG | 0.01 | 0.525*** | 0.336* | 0.608*** | 0.368** |
| CG | -0.178 | -0.15 | -0.553 | -0.363** | -0.429** |
| Gallic acid | -0.148 | 0.061 | -0.267 | -0.196 | -0.191 |
| Ellagic acid | 0.246 | -0.084 | 0.160 | -0.025 | -0.040 |
| Rutin | 0.424** | 0.420** | 0.175 | 0.265 | 0.270* |
| Taxifolin | 0.382** | 0.087 | 0.102 | -0.149 | 0.031 |
| Myricetin | 0.151 | 0.073 | 0.026 | 0.056 | 0.078 |
| Quercetin | -0.079 | -0.189 | -0.296* | -0.290* | -0.395** |
| Luteolin | 0.312* | -0.069 | 0.198 | -0.266 | 0.034 |
| Kaempferol | 0.228 | -0.020 | -0.213 | -0.399** | 0.132 |
| TGG | 0.034 | -0.077 | 0.245 | 0.088 | -0.005 |
| Caffeine | 0.282* | 0.502*** | 0.275* | 0.239 | 0.299* |
| Theobromine | -0.008 | 0.288* | 0.334* | 0.255 | 0.296* |
| Theophylline | -0.225 | -0.388** | -0.284* | -0.072 | -0.593*** |
| L-theanine | -0.405** | -0.500*** | -0.372** | -0.450** | -0.450** |
| L-arginine | -0.219 | -0.305* | -0.287* | 0.444** | -0.381** |
| L-aspartic acid | -0.008 | -0.130 | -0.182 | -0.375** | -0.382** |
| L-asparagine | -0.189 | -0.393** | -0.215 | -0.495*** | -0.372** |
| L-histidine | -0.446** | -0.516*** | -0.323* | -0.229 | -0.435** |
| L-isoleucine | -0.263 | -0.528*** | 0.304* | 0.320* | -0.499*** |
| L-glutamic acid | -0.125 | 0.025 | -0.075 | -0.041 | 0.087 |
| L-phenylalanine | 0.146 | -0.097 | 0.226 | -0.069 | 0.119 |
| L-tyrosine | -0.192 | -0.482*** | -0.392** | -0.479*** | 0.633*** |
| L-alanine | -0.498*** | -0.496*** | -0.618*** | -0.280* | -0.588*** |
| L-proline | -0.121 | -0.006 | 0.004 | -0.121 | -0.238 |
| L-methionine | -0.044 | 0.056 | -0.086 | 0.173 | -0.029 |
| L-cysteine | -0.163 | -0.344* | -0.207 | -0.477*** | -0.469*** |
| L-glycine | -0.369** | -0.444** | -290* | -0.062 | -0.378** |
| L-leucine | 0.195 | -0.313* | -0.023 | -0.524*** | -0.373** |
| L-serine | 0.126 | -0.103 | 0.084 | -0.131 | -0.163 |
| L-valine | 0.132 | -0.108 | 0.139 | -0.382** | -0.077 |
| L-threonine | -0.441** | 0.480*** | -0.551*** | -0.520*** | -0.455** |
| L-tryptophan | 0.047 | 0.09 | 0.038 | -0.225 | -0.124 |
| GABA | 0.129 | 0.384** | 0.019 | 0.226 | 0.107 |

FRAP: Ferric ion reducing antioxidant power; DPPH, DPPH free radical scavenging activity; ABTS, ABTS•+ scavenging activity; HSA, hydroxyl radical scavenging ability; SSA, superoxide anion radical scavenging ability.

Person correlation coefficient was determined by the bivariate correlation analysis using SPSS 20.0 software.

Significant levels: *** indicated *P* < 0.001, ** indicated 0.001 ≤ *P* < 0.01, *indicated 0.01 ≤ *P* < 0.05.


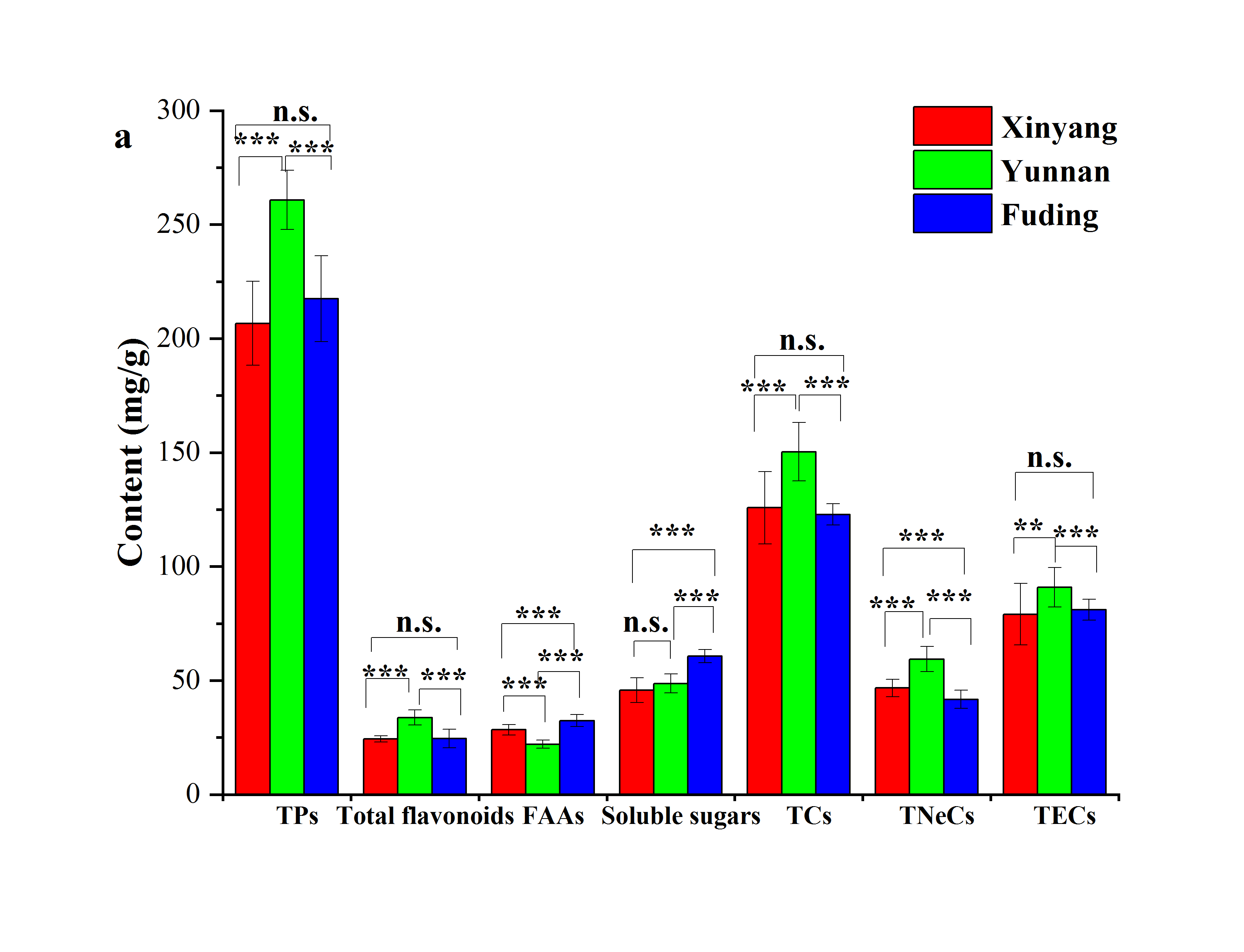

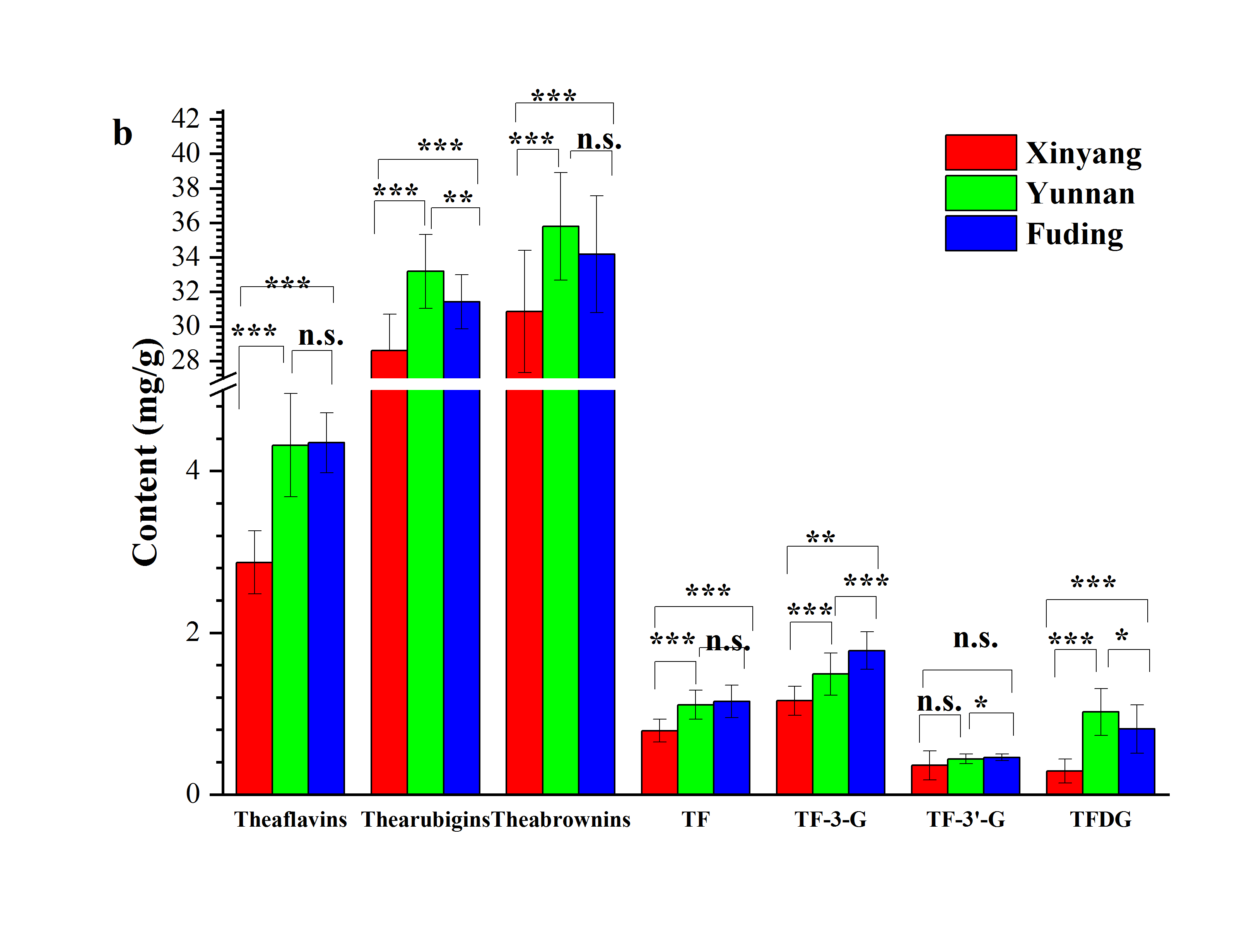


**Fig. S1.** Differences of seven quality components, three tea pigments and four theaflavins in white tea among Xinyang, Fuding and Yunnan regions evaluated by the independent-samples *t*-test.

TPs, tea polyphenols; FAAs, free amino acids; TCs, total catechins; TNeCs, total non-ester catechins; TECs, total ester catechins; TF, theaflavin; TF-3-G, theaflavin-3-gallate; TF-3'-G, theaflavin-3'-gallate; TFDG, theaflavine-3,3'-digallate.

TCs was the summation of eight catechins contents determined by HPLC. TNeCs was the summation of four non-ester catechins (i.e. C, EC, EGC and GC) contents. TECs was the summation of four ester catechins (i.e. ECG, GCG, EGCG and CG) contents. Statically significant difference levels were determined by independent-samples *t*-test: n.s., *P* ≥ 0.05; * indicated 0.01 ≤ *P* < 0.05; ** indicated 0.001 ≤ *P* < 0.01; *** indicated *P* < 0.001, respectively.


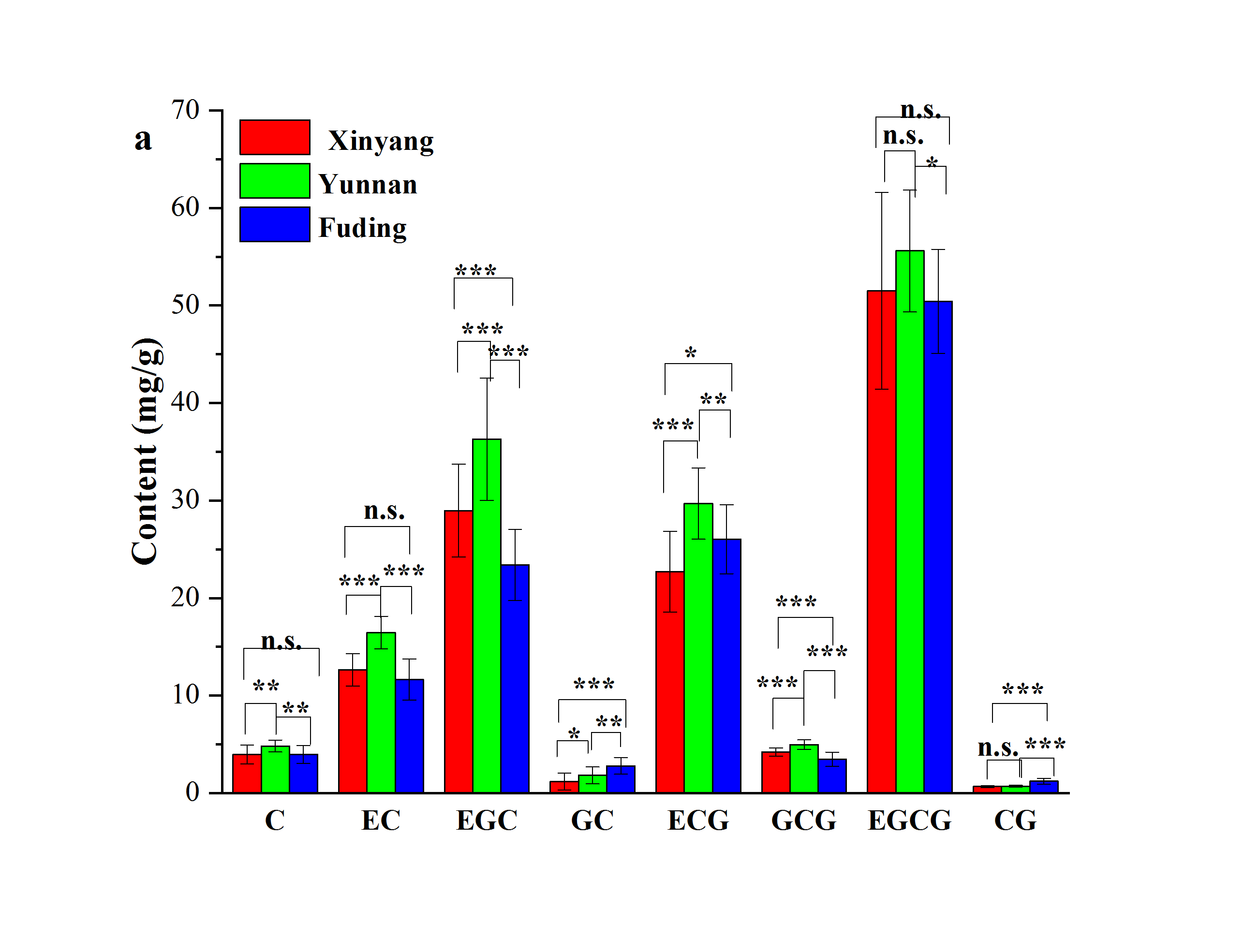

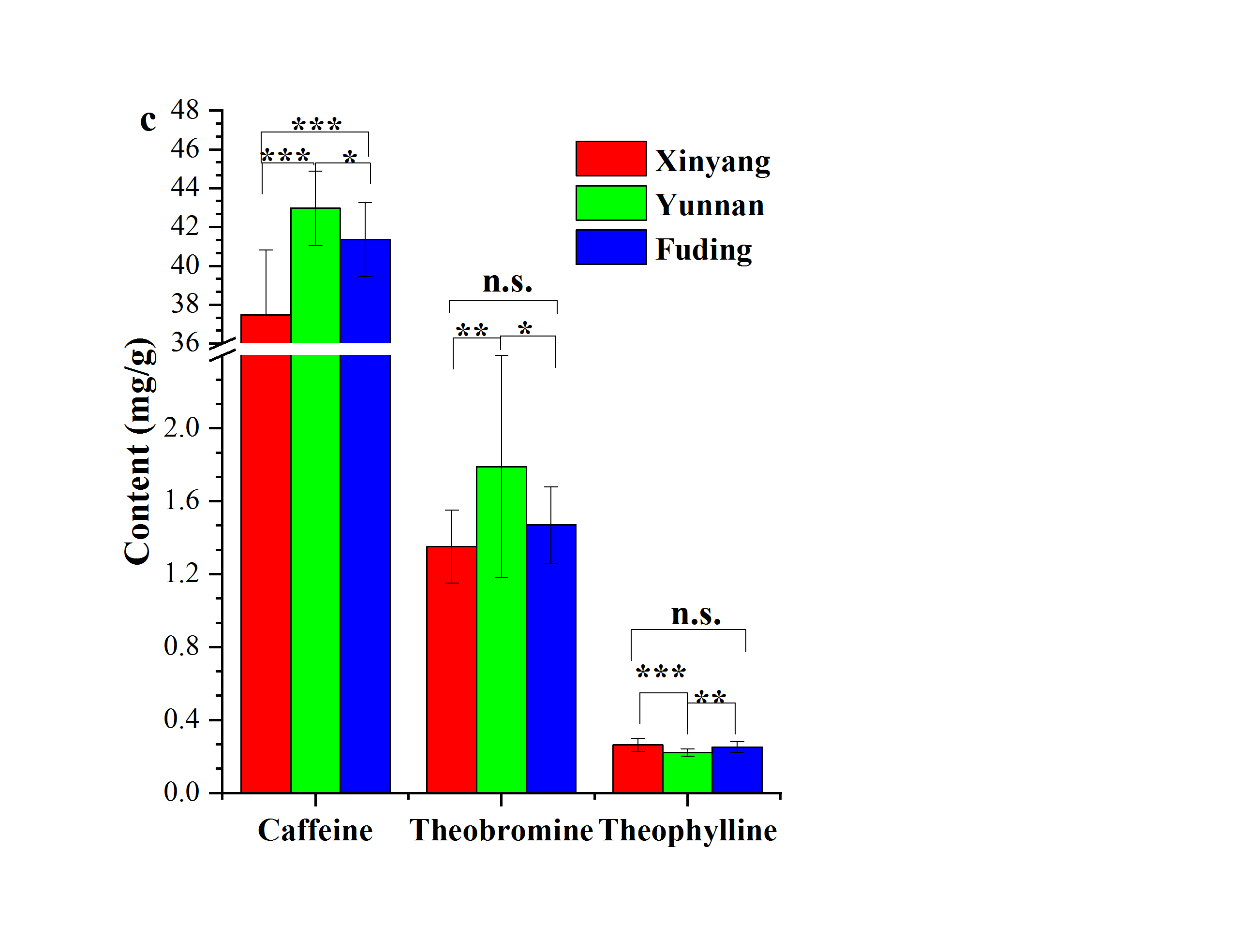


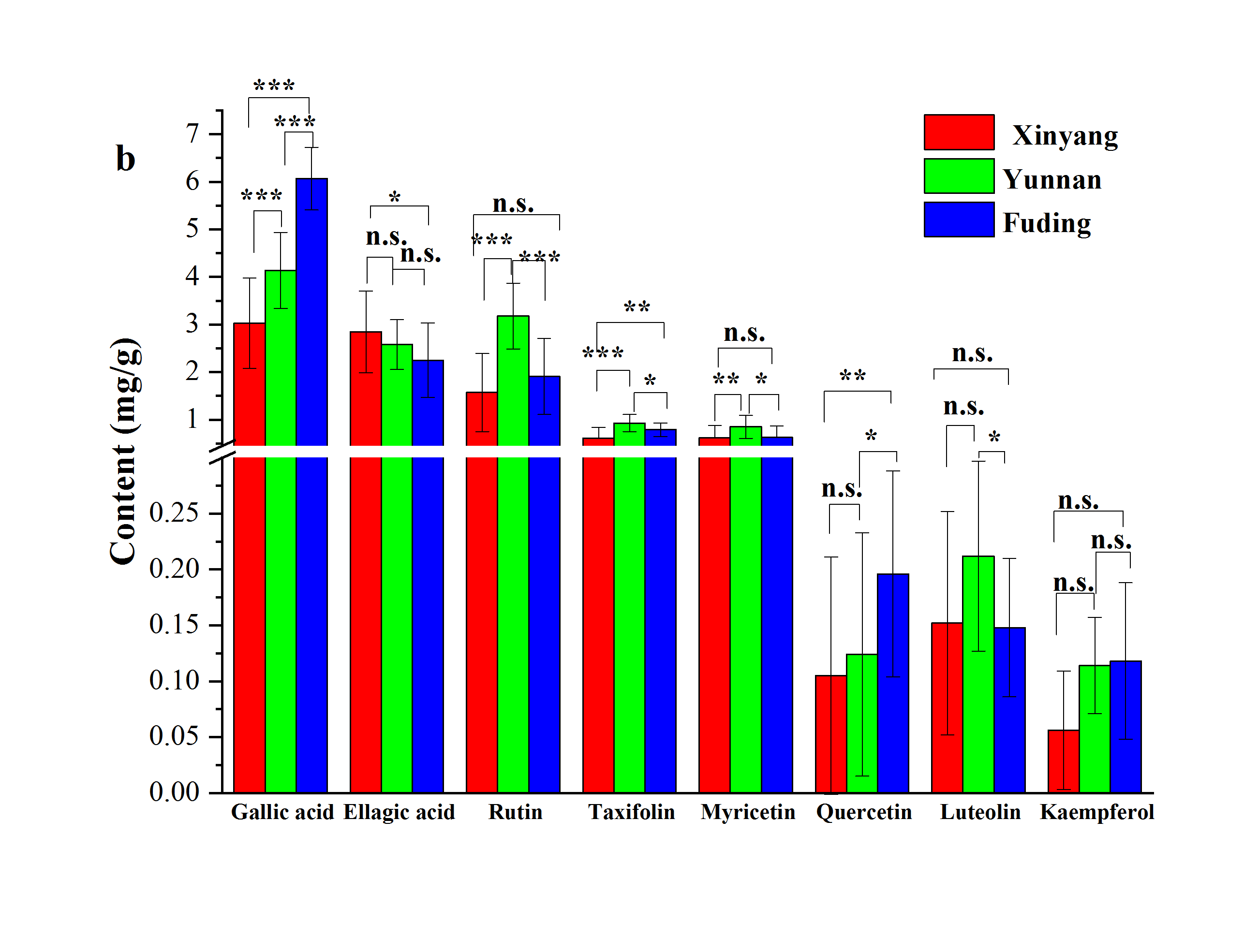


**Fig. S2.** Differences of eight catechins (a), two phenolic acids and six flavonoids (b), and three purine alkaloids (c) in white tea among Xinyang, Fuding and Yunnan regions evaluated by the independent-samples *t*-test.

Statically significant difference levels were determined by independent-samples *t*-test: n.s., *P* ≥ 0.05; * indicated 0.01 ≤ *P* < 0.05; ** indicated 0.001 ≤ *P* < 0.01; *** indicated *P* < 0.001, respectively.


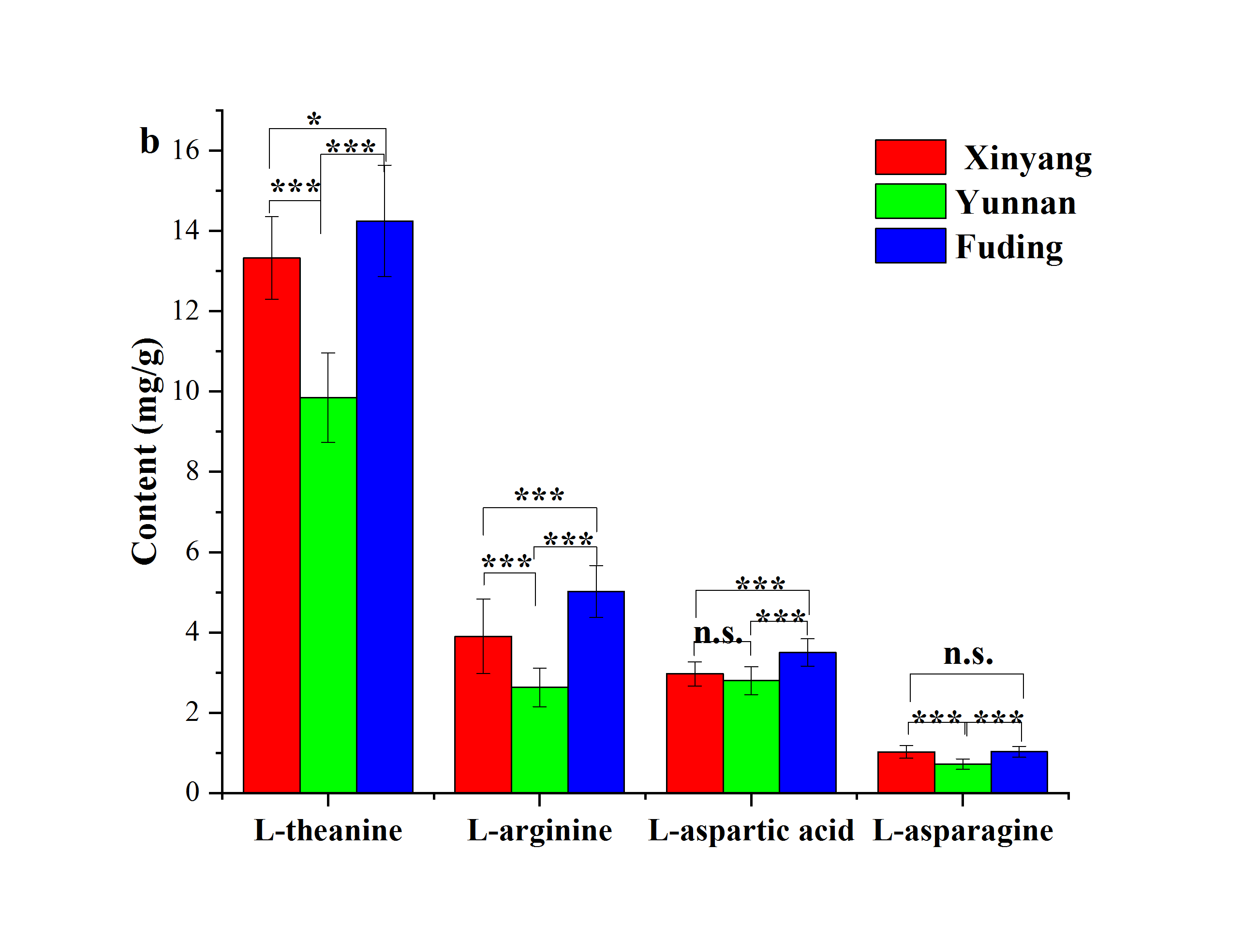

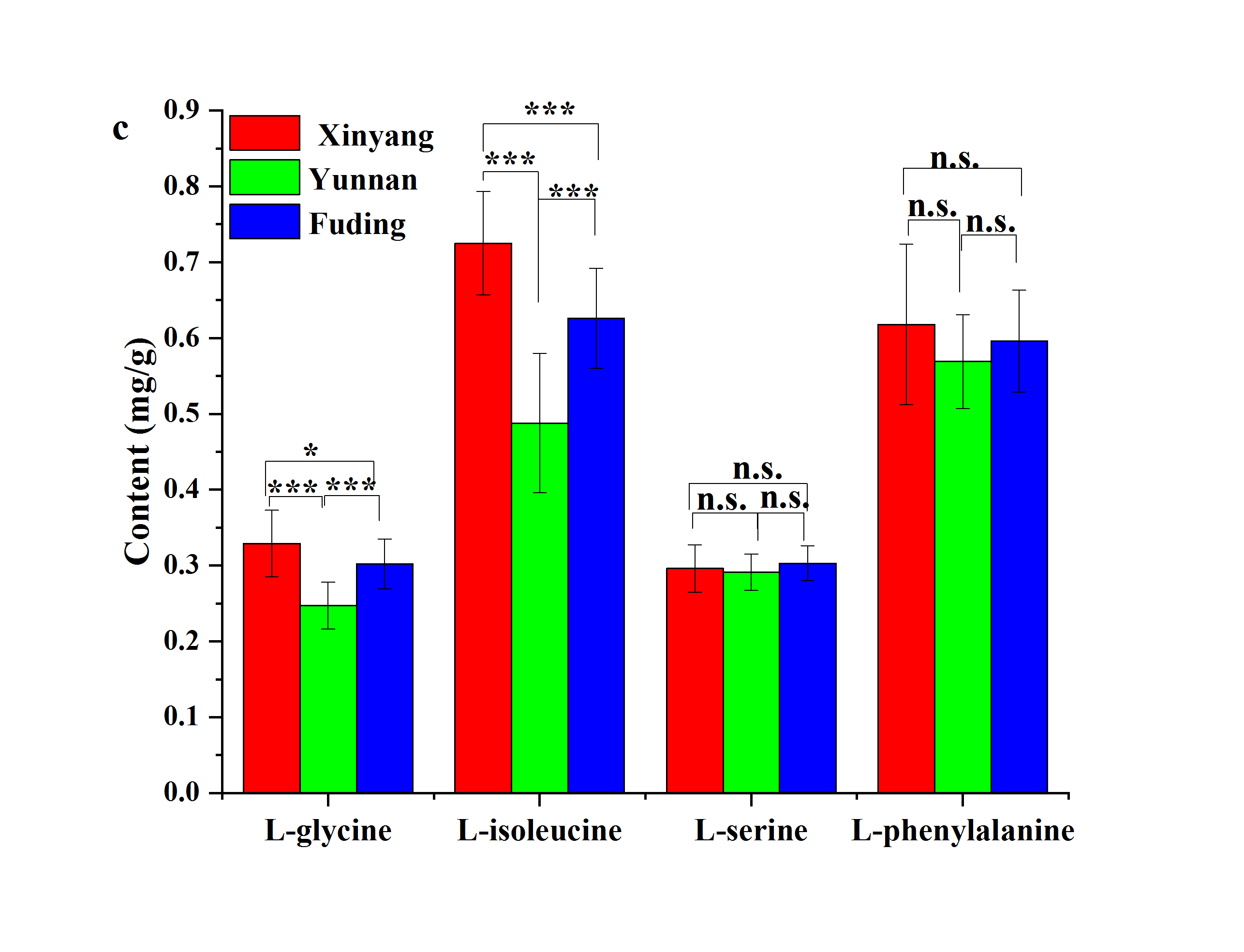

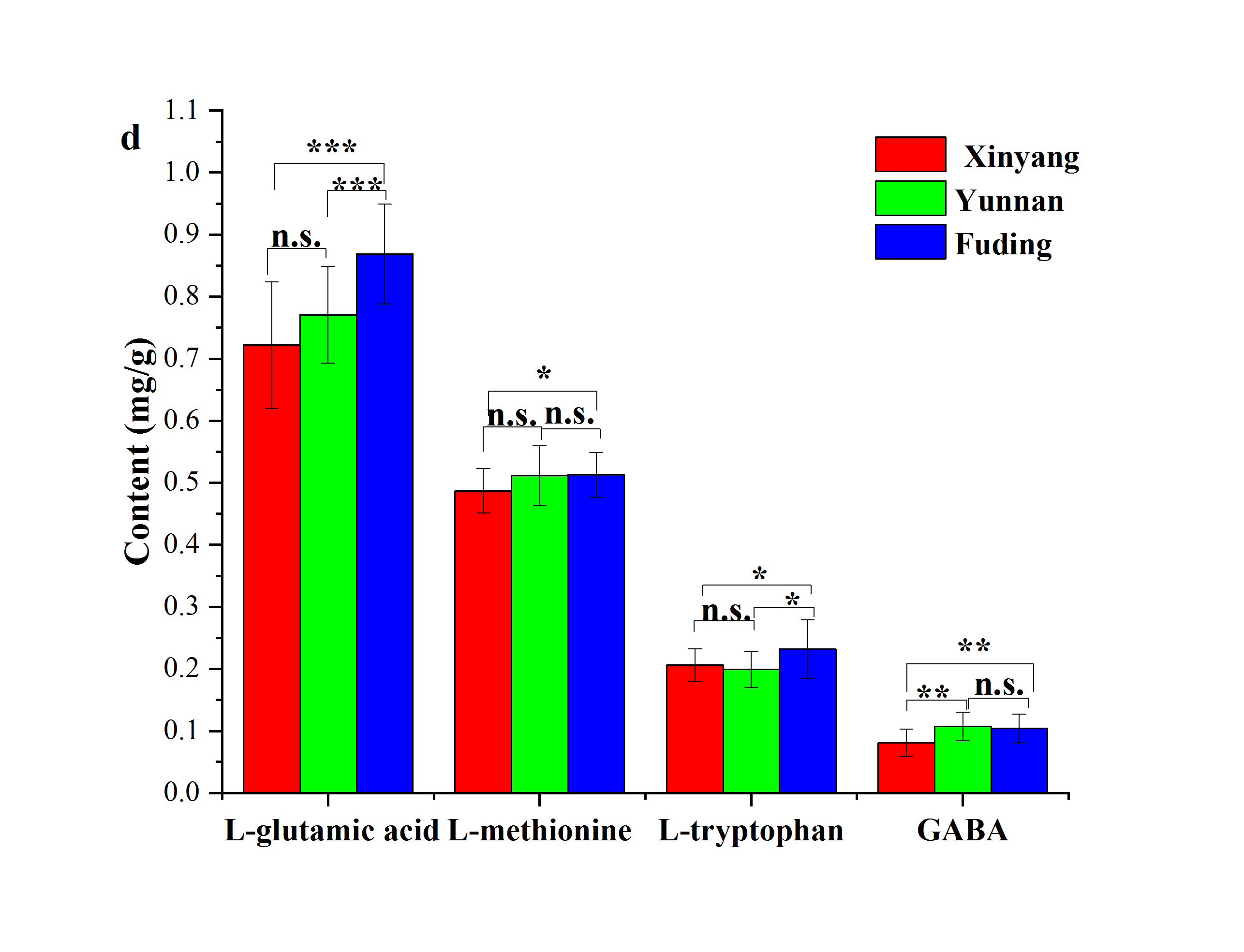

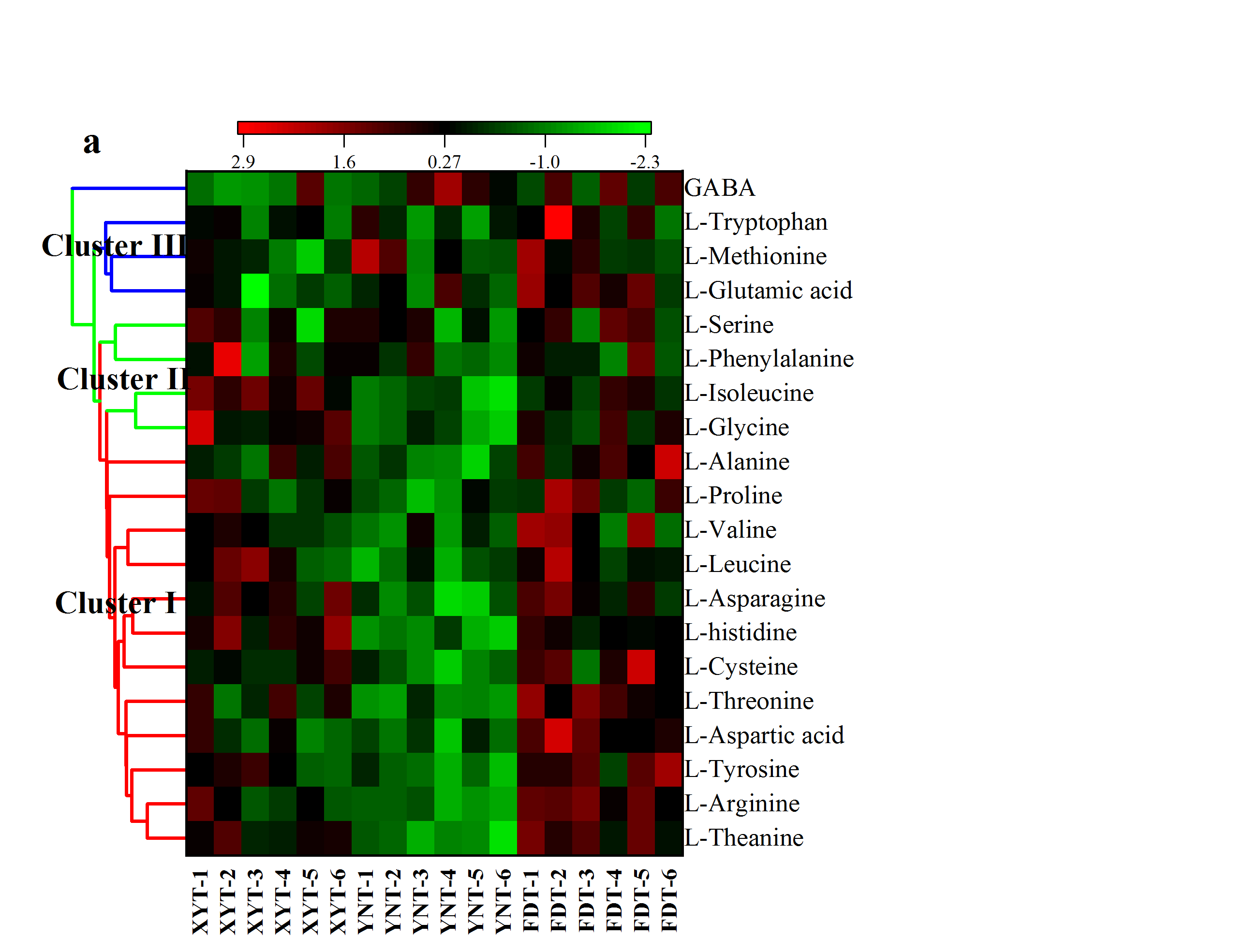


**Fig. S3.** Differences of nineteen amino acids and GABA in white tea among Xinyang, Yunnan and Fuding regions evaluated by heat map analysis (a) and the independent-samples *t*-test (b, c and d), respectively.

A: Heat map analysis indicated that these components could be assigned into three Clusters, i.e., Cluster I, Cluster II and Cluster III.

B: Yunnan white tea included relatively lower amino acids in Cluster I, such as L-theanine, L-arginie, L-aspartic acid and L-asparagine.

C: Xinyang white tea possessed relatively higher contents of components in Cluster II than Yunnan or Fuding white teas including L-glycine, L-isoleucine, L-serine and L-phenylalanine.

D: Yunan white tea contained relatively higher contents of components in Cluster III than Xinyang white tea, such as L-glutamic acid, L-methionine, L-tryptophan and GABA.

Statically significant difference levels were determined by independent-samples *t*-test: n.s., *P* ≥ 0.05; * indicated 0.01 ≤ *P* < 0.05; ** indicated 0.001 ≤ *P* < 0.01; *** indicated *P* < 0.001, respectively.
