# Supplementary material for: Geographical origin identification of Chinese white teas, and their differences in tastes, chemical compositions and antioxidant activities among three production regions
Source: Food Chem X. 2022 Nov 7;16:100504. doi: 10.1016/j.fochx.2022.100504 (PMC9743341; doi:10.1016/j.fochx.2022.100504)
Supplement: Supplementary data 2 [file mmc2.doc]

**Geographical origin identification of Chinese white teas, and their differences in tastes, chemical compositions and antioxidant activities among three production regions**

Cunqiang Ma ^a^, Bingsong Ma ^a,b^, Jiacai Wang ^b, d^, Zihao Wang ^c^, Xuan Chen ^a^, Binxing Zhou ^b*^, Xinghui Li ^a^

^a^ College of Horticulture, Nanjing Agricultural University, Nanjing, 210095, Jiangsu, China

^b^ College of Tea, Yunnan Agricultural University, Kunming, 650201,Yunnan, China

^c^ Henan Key Laboratory of Tea Comprehensive Utilization in South Henan, Tea College, Xinyang Agriculture and Forestry University, Xinyang, 464000, Henan, China.

^d^ China Tea (Menghai) Tea Industry Co. Ltd, Xishuangbanna, 666200, Yunnan, China.

1. **Materials and methods**

**2.1. Materials and reagents**

The 18 Bai Mudan sub-type of commercial white tea samples (**Table S1**) collected from Xinyang Region of Henan Province (named as XYT-1 to XYT-6), Fuding Region of Fujian Province (named as FDT-1 to FDT-6), and Lincang and Puer Regions of Yunnan Province (named as YNT-1 to YNT-6), were all made by the fresh tea-leaves with one bud and two leaves of the locally grown varieties/cultivars, such as Xinyang group (*Camellia* si*nensis* var. *sinensis* cv. Xinyang group), Fuding Dabaicha (*Camellia* si*nensis* var. *sinensis* cv. Fuding Dabaicha), Fuding Dahaocha (*Camellia* si*nensis* var. *sinensis* cv. Fuding Dahaocha), Jinggu Dabaicha (*Camellia sinensis* var. *assamica* cv. Jinggu Dabaicha) and Mengku Dayezhong (*Camellia sinensis* var. *assamica* cv. Mengku Dayezhong) in the spring season of 2020, and produced according to local processing technology. All processed tea samples were maintained at -20 °C with a moisture content below 6.5% for sensory evaluation and E-tongue measurement. In addition, tea powders (about 20 g) were collected through 40 mesh filtration for chemical determinations including phenolic compounds, purine alkaloids, theaflavins and amino acids, as well as antioxidant capacity test.

(+)-Catechin (C), (-)-epicatechin (EC), (-)-epigallocatechin **(**EGC), (-)-epicatechin gallate (ECG), (-)-gallocatechin gallate (GCG), (-)-epigallocatechin gallate (EGCG), (+)-gallocatechin (GC), (-)-catechin gallate (CG), gallic acid, ellagic acid and 1,4,6-tri-*O*-galloyl-β-D-glucose (TGG) (purity ≥98.0%) were purchased from Yuanye Bio-Technology Co., Ltd (Shanghai, China). Six flavonoids standards (purity ≥98.0%) namely quercetin, kaempferol, myricetin, taxifolin, luteolin and rutin were purchased from Must Bio-Technology Co., Ltd (Chengdu, Sichuan, China). Three purine alkaloids including caffeine, theobromine, theophylline, four theaflavins (i.e. theaflavin, theaflavin-3-gallate, theaflavin 3,3'-digallate and theaflavin-3'-gallate), nineteen amino acids (i.e. L-theanine, L-asparagine, L-glutamic acid, L-alanine, L-aspartic acid, L-argnine, L-cysteine, L-glycine, L-histidine, L-isoleucine, L-leucine, L-lysine, L-methionine, L-phenylalanine, L-proline, L-threonine, L-tryptophane, L-tyrosine and L-serine) and γ-aminobutyric acid (GABA) with a purity no less than 98.0% were purchased from Sigma-Aldrich Co., Ltd (St. Louis, MO, USA). Chromatographic-grade acetonitrile, methanol, 2-methoxyethanol and acetic acid were purchased from Aladdin Biological Co., Ltd (Shanghai, China).

**Table S1 Information of eighteen Bai mudan sub-type of white tea samples from three various origins.**

| No. | Production places | Cultivars | Size | Season |
| --- | --- | --- | --- | --- |
| XYT-1 | Shihe District Xinyang | Xinyang group | One bud two leaves | Spring |
| XYT-2 | Shihe District Xinyang | Xinyang group | One bud two leaves | Spring |
| XYT-3 | Shihe District Xinyang | Fuding Dabaicha | One bud two leaves | Spring |
| XYT-4 | Shihe District Xinyang | Fuding Dabaicha | One bud two leaves | Spring |
| XYT-5 | Shihe District Xinyang | Fuding Dabaicha | One bud two leaves in early development | Spring |
| XYT-6 | Shihe District Xinyang | Xinyang group | One bud two leaves | Spring |
| YNT-1 | Lincang of Yunnan | Mengku Dayezhong | One bud two leaves in early development | Spring |
| YNT-2 | Lincang of Yunnan | Mengku Dayezhong | One bud two leaves in early development | Spring |
| YNT-3 | Puer of Yunan | Jinggu Dabaicha | One bud two leaves | Spring |
| YNT-4 | Puer of Yunan | Jinggu Dabaicha | One bud two leaves | Spring |
| YNT-5 | Lincang of Yunnan | Mengku Dayezhong | One bud two leaves in early development | Spring |
| YNT-6 | Lincang of Yunnan | Mengku Dayezhong | One bud two leaves in early development | Spring |
| FDT-1 | Fuding of Fujian | Fuding Dabaicha | One bud two leaves | Spring |
| FDT-2 | Fuding of Fujian | Fuding Dabaicha | One bud two leaves | Spring |
| FDT-3 | Fuding of Fujian | Fuding Dahaocha | One bud two leaves | Spring |
| FDT-4 | Fuding of Fujian | Fuding Dahaocha | One bud two leaves in early development | Spring |
| FDT-5 | Fuding of Fujian | Fuding Dahaocha | One bud two leaves | Spring |
| FDT-6 | Fuding of Fujian | Fuding Dahaocha | One bud two leaves | Spring |

**2.2. Sensory evaluation**

The sensory panel composed of seven panelists selected from ten professional tea tasters, and the selection was based on evaluation performance of consistency and reliability (Qin et al., 2013). Sensory evaluation was based on five factors, including appearance (a), liquor color (b), aroma (c), and taste (d) and infused leaves (e) according to China National Institute of Standardization (CNIS) GB/T 23776-2018 (Gong et al., 2018). The evaluation procedures were as follows: a total of 100-150 g of tea samples were prepared for the evaluation of appearance; 3 g of samples were infused in 150 mL boiling water for 5 min; the liquor color, aroma, taste and infused leaves were evaluated after infused, respectively.

Total score was estimated as follows:

Total score = 20% × appearance (a) + 10% × liquor color (b) + 30% × aroma (c) + 30% × taste (d) + 10% × tea-leaves residues (e).

**2.3. E-tongue measurements for taste evaluation**

The E-tongue (SA402B, INSENT, Japan) comprised six taste sensors, such as umami (1-AAE), saltiness (2-CTO), sourness (3-CAO), bitterness(5-COO), astringency (6-AE1) and sweetness (5-GL1), and reference electrode (R) for the sensory taste evaluation including umami, saltiness, sourness, bitterness, astringency and sweetness. The taste sensor consisted of a multi-channel bionic lipid membrane, an Ag/AgCl electrode, and an internal cavity filled with a 3.3 M KCl aqueous solution saturated with AgCl. E-tongue tests were carried out with 30 mM KCl and 0.3 mM tartaric acid aqueous solution as the reference solution. Based on GB/T 23776-2018 methodology of sensory evaluation of tea, the uniform proportion of tea to water applied in the measurements is 1:50 (g/mL). A total of 3 g of each tea sample was infused with 150 mL distilled boiling water for 5 min to obtain a tea soup, which was cooled to indoor temperature about 25 °C for the potentiometric measurements (Xu et al., 2019). This experiment consisted of three measurement phases: sample detection phase (120 s), “aftertaste” detection phase (40 s), and cleaning phase (10 s). During sample detection phase, the data from 110 to 120 s were collected to calculate average value to be final result. Then, the taste strength of tea soup was measured in 4 times to get the average value, and each sample was measured in triplicate on the same day.

**2.4. Determinations of seven main quality components by spectrophotometer**

Moisture content and water extracts content of each white tea were measured according to the national standard methods of GB 5009.3-2016 and GB/T 8305-2013 established by China National Institute of Standardization (CNIS). The total content of tea polyphenols was determined on 765 nm a TU-1901 by ultraviolet-visible (UV-Vis) spectrophotometer (Puxi Technologies, Beijing, China) through Foline-Ciocalteu Phenol Reagent (Wang, Zheng et al., 2021). Total flavonoids content was measured by NaNO_2_-AlCl_3_ colorimetric method on 510 nm with rutin as the standard using the UV-Vis spectrophotometer (Tong, Liu, Kang, Zhang & Kang, 2019). Total soluble-sugars content was measured on 620 nm by an UV-Vis spectrophotometer method with anthrone, which has been described in our previous studies of Zhou, Ma et al., (2020a; 2020b). The total content of free amino acids was measured on 570 nm by the UV-Vis spectrophotometer through ninhydrin assay (Zhou, Ma et al., 2020a; 2022b). Three tea pigments (i.e. theaflavins, thearubigins and theabrownins) were systematically analyzed on 380 nm using the UV-Vis spectrophotometer, which has been described in the previous report by Zhou et al. (2022). Each tea sample was determined with three replications.

**2.5. Seventeen phenolic compounds and three purine alkaloids contents determined by high-performance liquid chromatography (HPLC)**

The tea powder (1,000 mg) was extracted with 44 mL ethyl alcohol-hydrochloric acid (40:4 v/v) for 90 min at 85 °C, and diluted with about 6 mL methyl alcohol to a volume of 50 mL. And then, about 2 μL of tea extraction after filtration through 0.45 μm nylon membrane filter was injected into an Agilent 1200 series HPLC system (Agilent Technologies, Santa Clara, CA, USA) for the determination of seventeen phenolic compounds including eight catechins, six flavonoids, two phenolic acids and TGG, as well as three purine alkaloids contents in eighteen white tea samples. The an Agilent 1200 series HPLC system was comprised of Poroshell 120 EC-C18 chromatogram column (100 mm × 4.6 mm, 2.7 μm; Agilent Technologies, Santa Clara, CA, USA) and a C18 guard column (10 mm × 4.6 mm, 5 μm; Phenomenex, Torrance, CA, USA) with solvent A (5% acetonitrile and 0.261% ortho-phosphoric acid water solution) and solvent B (80% methanol solution) as mobile phase for HPLC separation (Nian et al., 2019). The gradient was programmed as follows: from 0–16 min solvent B was increased from 10 to 45%; from 16–22 min solvent B was increased to 65%; from 22–25.9 min, solvent B was increased to 100%; from 25.9-29 min, solvent B kept at 100%; from 29-30 min, solvent B was decreased to 10%; from 30-36 min, solvent B kept at 10% (Zhou, Wang et al., 2022; Ma, Wang et al., 2022). The flow rate was 0.8 mL/min. The column temperature was set at 30℃. The detection wavelength was 280 nm (0-20 min) and 360 nm (20-36 min) using a wavelength switching detection method. Internal standard method was used to aid in the identification of bioactive compounds. Quantitative analysis was carried out based on the linear regression equation of twenty standards. Each tea sample was determined with three replications. The linear regression equation with a high correlation coefficient (R^2^ > 0.990) were established for quantitative determination.

**2.6. Four theaflavins in white tea determined by HPLC**

The 200 mg tea powder was extracted with 5 mL 70% (v/v) methanol solution at 70 °C for 10 min. Repeat extraction was carried out to obtain a metered volume of 10 mL after centrifugation at 3500 rpm for 5 min. The tea extract was filtered by 0.45 μm nylon membrane filter and 5 μL filter liquor was injected into Agilent 1100VL series HPLC system (Agilent Technologies, Santa Clara, CA, USA) for the quantitative analysis of theaflavin, theaflavin-3-gallate, theaflavin 3,3'-digallate and theaflavin-3'-gallate. An Agilent SB-Aq C18 reversed-phase chromatogram column (250 × 4.6 mm, 5 μm) with Solvent A (90 mL acetonitrile and 20 mL acetic acid with 20 mg EDTA-2Na in 1000 mL water solution) and solvent B (800 mL methanol and 20 mL acetic acid with 20 mg EDTA-2Na in 1000 mL water solution) as the mobile phases were prepared for HPLC separation. The gradient was programmed as follows: from 0-2 min, solvent A kept at 100%; from 2-5 min, solvent A linearly decreased from 100% to 95%; from 5-10 min, solvent A linearly decreased from 95% to 90%; from 10-20 min, solvent A linearly decreased from 90% to 70%; from 20-25 min, solvent A linearly decreased from 70% to 65%; from 25-30 min, solvent A linearly increased from 65% to 70%; from 30-35 min, solvent A linearly increased from 70% to 100%. The whole HPLC separation detected at 278 nm remained a flow rate of 0.7 mL/min at a column temperature of 35℃. Three replications were carried to acquire reliable data. The analytical curves (R^2^ > 0.990) were establish to calculate the quantitative contents.

**2.7. Nineteen amino acids and GABA contents determined by amino acid analyzer**

In this work, nineteen free amino acids (FAAs) including L-theanine, L-asparagine, L-alanine, L-aspartic acid, L-argnine, L-cysteine, L-glutamic acid, L-glycine, L-histidine, L-isoleucine, L-leucine, L-methionine, L-phenylalanine, L-proline, L-threonine, L-tryptophane, L-tyrosine, L-serine and L-valine, and γ-aminobutyric acid (GABA) level in the each white tea sample was determined by a S-433D amino acid analyzer (Sykam Technologies, Munich, Bavaria, Germany) according to GB/T 30987-2014 with a LCA K07/Li cation separation column (150 × 4.6 mm, 3 μm) using ion-exchange chromatography (IEC) after ninhydrin derivations (Tan, Xu, Zhao, Sun, & Tan, 2014). The tea powder (2,000 mg) were extracted with 250 mL boiling water in 95 ℃ water for about 10 min and tea extracts were diluted to 250 mL after suction filtration (Ma et al., 2021; Zhou, Ma et al., 2022a). The solution was filtered by 0.45 μm water membrane filter for the detection. The flow rates of buffer solution and ninhydrin solution were 0.45 mL/min and 0.25 mL/min, and 50 μL of extraction was injected, respectively. The reaction column and reactor temperature were set at 58 °C and 130 °C. The detection wavelength was 570 nm and 440 nm, respectively. Based on relevant standard curves, the linear regression equations with a high correlation coefficient (R^2^ > 0.999) were established for the quantitative determination of nineteen amino acids and GABA in eighteen white tea samples. Each white tea sample was determined with three replications.

**2.8. *In vitro* antioxidant capacity evaluation by five various assays**

The *in vitro* antioxidant activities were evaluated by five various assays including ferric ion reducing antioxidant power (FRAP), DPPH free radical scavenging activity (DPPH), ABTS•+ scavenging activity (ABTS), hydroxyl radical scavenging ability (HSA) and superoxide anion radical scavenging ability (SSA), respectively, which has been described in our reports of Zhou, Wang et al., (2022) and Ma, Wang et al., 2022.

2.8.1. FRAP assay

The tea powder (50 mg) mixed with 1 mL 80% (v/v) ethanol water was extracted by the ultrasonic producer at 60 °C for 30 min with a power of 200~300V. The supernatant was collected after centrifugation at 12700×g for 10 min and determined on 590 nm by Multiskan FC series microplate reader (Thermo Scientific, Waltham, MA, USA). The FRAP of each white tea sample was calculated with trolox as the standard through the calibration curve as follows: Y_1_=0.0972X_1_+0.0042 (R^2^=0.9997). (X_1_ is the amount of trolox, nmol; Y_1_ is OD_590_, respectively).

2.8.2. DPPH assay

The tea powder (50 mg) mixed with 1 mL 80% (v/v) methanol extract was extracted by tissue homogenate in an ice bath. The supernatant was collected after centrifugation at 12700×g for 10 min, and measured on 517 nm by the microplate reader. DPPH radical scavenging activity of each white tea sample was calculated with trolox as the standard through the calibration curve as follows: Y_2_=2.8486X_2_+0.7084 (R^2^=0.9991). (X_2_ is the concentration of trolox, μg/mL; Y_2_ is the DPPH radical scavenging rate, %).

2.8.3. ABTS assay

The tea powder (50 mg) mixed with 1 mL 80% (v/v) methanol extract was extracted by tissue homogenate in an ice bath. The supernatant was collected after centrifugation at 12700×g for 10 min, and measured on 734 nm. With trolox as the standard, ABTS radical cation scavenging activity of each white tea sample was calculated through the calibration curve as follows: Y_3_=0.5042X_3_-1.4213 (R^2^=0.9971). (X_3_ is the concentration of trolox, μg/mL; Y_3_ is the ABTS radical cation scavenging rate, %).

2.8.4. HSA assay

The tea powder (100 mg) was extracted by tissue homogenate in an ice bath with 1 mL distilled water. After centrifugation at 12700×g for 10 min, the absorbance at 510 nm of the supernatant was determined by the microplate reader to calculate the rate of HSA based on the equation (1):

(1)

2.8.5. SSA assay

The tea powder (100 mg) was extracted by tissue homogenate in an ice bath with 1 mL extracting solution. After centrifugation at 12700×g for 10 min, the absorbance at 570 nm of the supernatant was determined by the microplate reader to calculate the rate of SSA based on the equation (2):

(2)

**2.8. Statistical analysis**

Each tea samples was determined with three replications and all data were present by mean value ± standard deviation (SD). Principal component analysis (PCA), hierarchical cluster analysis (HCA) and partial least squares-discriminant analysis (PLS-DA) were performed with 54 selected objects (18×3) by Origin 9.0 software (Hampton, MA, USA). One-way analysis of variance (ANOVA) by using Duncan`s multiple comparative analysis method (for ≥ three groups), independent-samples *t*-test (for two groups) and the bivariate correlation analysis between relevant chemical components to 6 taste factors and antioxidant activities detected by 5 various assays were carried out performed with IBM SPSS 20.0 software (Chicago, IL, USA). The relevant heat maps were performed by Origin 9.0 software (Hampton, MA, USA). The characteristic components were selected with a variable importance in the projection over 1.0 (VIP > 1.0) in PLS-DA and P-value below 0.05 (P < 0.05) in ANOVA for the geographical identification of Chinese white teas.

**References**

Gong, S. Y., Zhao, Y. X., Lu, C. Y., Liu, X., Guo, Y. L., Zhang, Y. B., … Dang, Q. Y. (2018). Methodology for sensory evaluation of tea GB/T 23776. (China Standards Press, Beijing, China).

Ma, B., Wang, J., Xu, C., Wang, Z., Yin, D., Zhou, B., & Ma, C. (2022). Interrelation analysis between phenolic compounds and *in vitro* antioxidant activities in Pu-erh tea. *LWT- Food Science and Technology*, *158*, 113117.

Ma, C., Li, X., Zheng, C., Zhou, B., Xu, C., & Xia T. (2021). Comparison of characteristic components in tea-leaves fermented by *Aspergillus pallidofulvus* PT-3, *Aspergillus sesamicola* PT-4 and *Penicillium manginii* PT-5 using LC-MS metabolomics and HPLC analysis. *Food Chemistry*, *350*, 129228.

<https://doi.org/10.1016/j.foodchem.2021.129228>

Nian, B., Chen, L., Yi, C., Shi, X., Jiang, B., Jiao, W., ... Zhao, M. (2019). A high performance liquid chromatography method for simultaneous detection of 20 bioactive components in tea extracts. *Electrophoresis*, *40*(21), 2837-2844.

Qin, Z., Pang, X., Chen, D., Cheng, H., Hu, X., & Wu, J. (2013). Evaluation of Chinese tea by the electronic nose and gas chromatography-mass spectrometry: correlation with sensory properties and classification according to grade level. *Food Research International*, *53*, 864-874.

Tan, H., Xu, W., Zhao, A., Sun, D., & Tan, F. (2014). *Determination of free amino acids in plants GB/T 30987*. Beijing, China: China Standards Press.

Tong, T., Liu, Y. J., Kang, J., Zhang, C. M., & Kang, S. G. (2019). Antioxidant activity and main chemical components of a novel fermented tea. *Molecules,* *24*, 2917.

Wang, Z., Zheng, C., Ma, C., Ma, B., Wang, J., Zhou, B., & Xia, T. (2021). Comparative analysis of chemical constituents and antioxidant activity in tea-leaves microbial fermentation of seven tea-derived fungi from ripened Pu-erh tea. *LWT-Food Science and Technology,* *142*, 111006.

Xu, S., Wang, J. J., Wei, Y., Deng, W. W., Wan, X., Bao, G. H., ... Ning, J. M. (2019). Metabolomics based on UHPLC-Orbitrap-MS and Global Natural Product Social Molecular Networking reveals effects of time scale and environment of storage on the metabolites and taste quality of raw Pu-erh tea. *Journal of Agricultural and Food Chemistry,* 67(43), 12084-12093.

Zhou, B., Ma, C., Wu, T., Xu, C., Wang, J. & Xia, T. (2020a), Classification of raw Pu-erh teas with different storage time based on characteristic compounds and effect of storage environment. *LWT-Food Science and Technology,* *133*, 109914.

Zhou, B., Ma C., Ren, X., Xia, T., Zheng, C., & Liu, X. (2020b). Correlation analysis between filamentous fungi and chemical compositions in a pu-erh type tea after a long-term storage. *Food Science & Nutrition,* *8*(5), 2501-2511.

Zhou, B., Ma, B., Ma, C., Xu, C., Wang, J., Wang, Z., ... Xia, T. (2022a). Classification of Pu-erh ripened teas and their differences in chemical constituents and antioxidant capacity. LWT-Food Science and Technology, 153, 112370.

Zhou, B., Ma, B., Xu, C., Wang, J., Wang, Z., Huang, Y., & Ma, C. (2022b). Impact of enzymatic fermentation on taste, chemical compositions and in vitro antioxidant activities in Chinese teas using E-tongue, HPLC and amino acid analyzer. LWT-Food Science and Technology, 163, 113549.

Zhou, B., Wang, Z., Yin, P., Ma, B., Ma, C., Xu, C., ... Xia, T. (2022). Impact of prolonged withering on phenolic compounds and antioxidant capability in white tea using LC-MS-based metabolomics and HPLC analysis: Comparison with green tea*. Food Chemistry*, 368, 130855.
